# Alpha-lipoic acid as a pleiotropic compound with potential therapeutic use in diabetes and other chronic diseases

**DOI:** 10.1186/1758-5996-6-80

**Published:** 2014-07-28

**Authors:** Marilia Brito Gomes, Carlos Antonio Negrato

**Affiliations:** Department of Internal Medicine, Diabetes Unit, State University Hospital of Rio de Janeiro, Avenida 28 de Setembro, 77, 3° andar CEP 20.551-030, Rio de Janeiro, Brazil; Department of Internal Medicine, Bauru’s Diabetics Association, 17012-433 Bauru, São Paulo, Brazil

**Keywords:** Alpha-lipoic acid, Biochemical action, Diabetes mellitus, Chronic diseases

## Abstract

Alpha-lipoic acid is a naturally occurring substance, essential for the function of different enzymes that take part in mitochondria’s oxidative metabolism. It is believed that alpha-lipoic acid or its reduced form, dihydrolipoic acid have many biochemical functions acting as biological antioxidants, as metal chelators, reducers of the oxidized forms of other antioxidant agents such as vitamin C and E, and modulator of the signaling transduction of several pathways. These above-mentioned actions have been shown in experimental studies emphasizing the use of alpha-lipoic acid as a potential therapeutic agent for many chronic diseases with great epidemiological as well economic and social impact such as brain diseases and cognitive dysfunctions like Alzheimer disease, obesity, nonalcoholic fatty liver disease, burning mouth syndrome, cardiovascular disease, hypertension, some types of cancer, glaucoma and osteoporosis. Many conflicting data have been found concerning the clinical use of alpha-lipoic acid in the treatment of diabetes and of diabetes-related chronic complications such as retinopathy, nephropathy, neuropathy, wound healing and diabetic cardiovascular autonomic neuropathy. The most frequent clinical condition in which alpha-lipoic acid has been studied was in the management of diabetic peripheral neuropathy in patients with type 1 as well type 2 diabetes. Considering that oxidative stress, a imbalance between pro and antioxidants with excessive production of reactive oxygen species, is a factor in the development of many diseases and that alpha-lipoic acid, a natural thiol antioxidant, has been shown to have beneficial effects on oxidative stress parameters in various tissues we wrote this article in order to make an up-to-date review of current thinking regarding alpha-lipoic acid and its use as an antioxidant drug therapy for a myriad of diseases that could have potential benefits from its use.

**Electronic supplementary material:**

The online version of this article (doi:10.1186/1758-5996-6-80) contains supplementary material, which is available to authorized users.

## Introduction

Alpha-lipoic acid (ALA) also known as thioctic acid (TA) and 1,2 dithiolane -3-pentanoic acid, is a naturally occurring substance, that is essential for the function of different enzymes of oxidative metabolism [1–3]. ALA was discovered in 1937 by Snell [4] but only in 1951 it was isolated by Reed [5]. The first clinical use of ALA has been described in Germany in 1959 for the treatment of acute poisoning with *amanita phalloides* commonly known as death cap [from mushrooms] a deadly poison widely distributed in Europe Moreover, soon after, the same authors described its utility in treating neuropathic complaints [6]. Nowadays it is believed that ALA or its reduced form, dihydrolipoic acid (DHLA) have many biochemical functions acting as biological antioxidants, as metal chelators, reducing the oxidized forms of other antioxidant agents such as vitamin C and E and glutathione (GSH), and modulating the signaling transduction of several pathways, like insulin and nuclear factor kappa B (NFkB) [1]. ALA has also shown to improve endothelial dysfunction [7] and to reduce oxidative stress post exercise training [8]; it also protects against the development of atherosclerosis and inhibits the progression of an already established atherosclerosis plaque [9, 10]. These above-mentioned actions have emphasized the use of ALA as a potential therapeutic agent for many chronic diseases with great epidemiological as well economic and social impact such as diabetes mellitus (DM) and its complications [11, 12], hypertension [13], Alzheimer’s disease (AD) [14], Down syndrome [15], cognitive dysfunction and some types of cancer [16]. Currently the use of ALA as a dietary supplement is growing in many aspects of medical and nutritional management of patients.

Considering the pleiotropic actions of ALA or DHLA in so many different organs and systems, in many different ways, this review has the objective to improve the clinical and biochemical understanding of its potential use in routine clinical care for a large spectrum of pathologies.

## Synthesis, biochemical properties, absorption and bioavailability

ALA is commonly found in dietary components such as vegetables (spinach, broccoli, tomato) and meats, mainly viscera and also in many dietary supplements. ALA can be also synthesized through enzymatic reactions in plants and animals’ mitochondria from octanoic acid and cysteine (as a sulfur donor) [17, 18]. As a sulfur containing substance, ALA is considered a thiol compound. Mammalian cells can synthesize ALA through the action of mitochondria lipoic acid synthase (LASY) which can be down-regulated in different clinical conditions [18].

**Figure 1:**
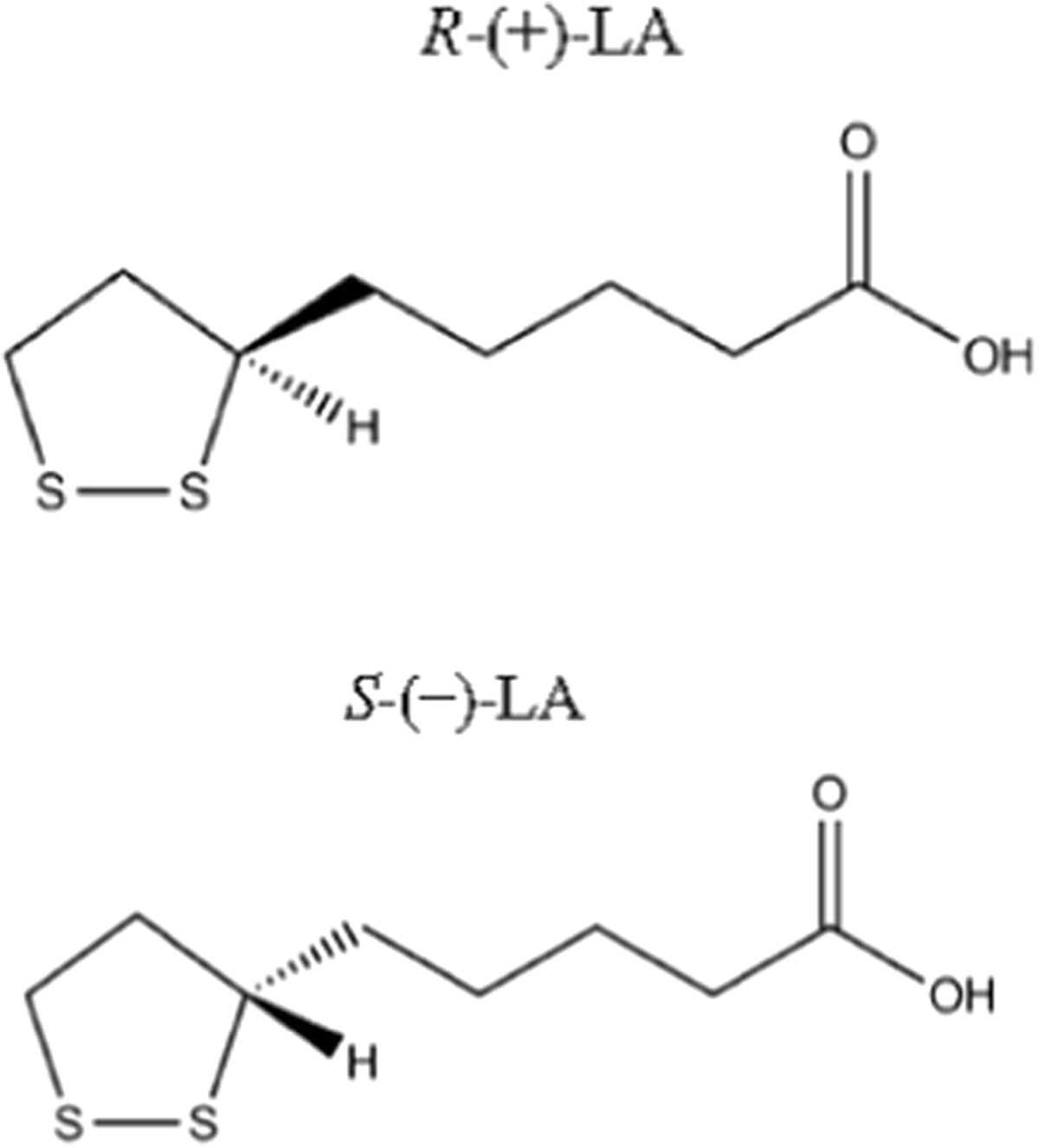
**Enantiomers (R and S) of lipoic acid.**

ALA exists in two enantiomeric (optical isomers) forms, R and S, (Figure 1) being the R isoform an essential cofactor for mitochondrial enzymes of oxidative metabolism since it is joined in amide linkage to €-amino group of lysine residues (lipoamide) [17]. The following enzymes use R-ALA as a cofactor: pyruvate dehydrogenase (PDH), branched chain α-keto-acid dehydrogenase (KDH) and α-ketoglutarate dehydrogenase (KGDH) [18, 19]. Pyruvate dehydrogenase is a multienzyme complex, composed by three enzymes, which catalyze in three steps the irreversible oxidative decarboxylation of pyruvate into acetyl coenzyme A (acetyl-CoA), which is a component of the citric acid cycle [19]. The other above-mentioned enzymes also catalyze the oxidative decarboxylation of other α-keto-acid such as α-ketoglutarate, valine, leucine, isoleucine. R-ALA is also a cofactor of glycine cleavage system which degrades glycine into pyruvate [20].

The absorption and bioavailability of ALA have been studied mainly from dietary supplements where ALA exists as an admixture of R-ALA and S-ALA. In general, the absolute bioavailability of both ennatiomers is not greater than 40% which decreases with food intake [12]. Therefore, ALA must be taken 30 min before meals. Some experimental studies have shown that R-ALA has greater biopotency in several metabolic pathways compared to S-ALA [21].

After oral intake, ALA is absorbed by the gastrointestinal tract and is transported to different organs such as brain because it has the potential of freely cross the blood–brain barrier [3]. Independently of the original sources (diet or nutritional supplements) ALA is reduced to DHLA and metabolized in the liver in different metabolites like bisnorlipoate and, tetranorlipoate, and has renal excretion.

So far, some systems have been associated with the cellular transport of ALA like sodium dependent transport, a transmembrane protein which is produced by the SLC5A6 gene that also translocates other vitamins and cofactors. Both transporters are also responsible for ALA intestinal uptake [22].

## Antioxidant properties

**Figure 2:**
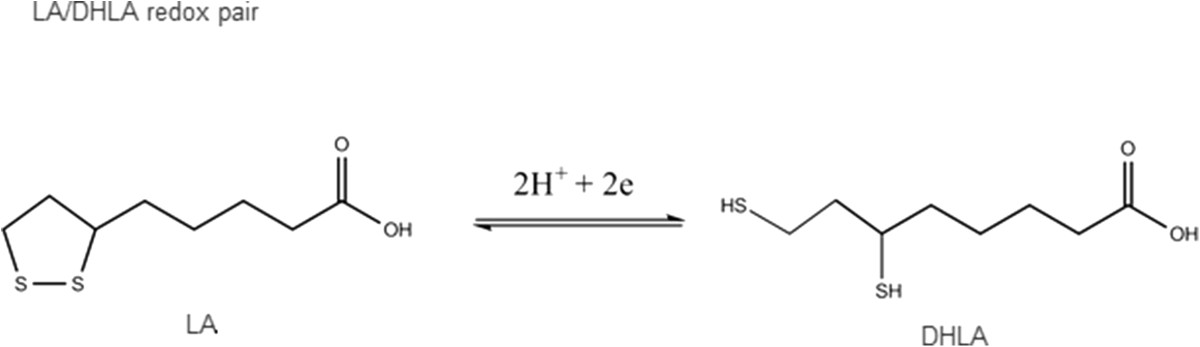
**Chemical structure of alpha lipoic acid (ALA) and its reduced from dihydrolipoic acid (DHLA).**

ALA and its reduced form DHLA, are considered powerful natural antioxidant agents with a scavenging capacity for many reactive oxygen species [23, 24]. The chemical structure of both compounds is showed in Figure 2. It is important to note that there is no agreement about the specific scavenging capacity of each form [23–31]. Although it is beyond of the scope of this review, for instances it was described a different scavenging ability of ALA and DHLA on aqueous and membrane phase of an experimental study which means that the environment could be an important factor for determining its scavenging capacity (24). A summary of these data are presented in Table 1. ALA/DHLA have some important advantages over other antioxidant agents such as vitamin E and C, because they have amphiphilic properties that confer their antioxidant actions in the membrane as well as in the cytosol [23].

**Table 1:** **Antioxidant action of ALA and DHLA upon reactive oxygen species and references**

Reactive oxygen species	ALA	DHLA
Hydrogen peroxide	Yes (12)	Yes (12)
(H_2_O_2_)	No (23)	No (23)
Superoxide		
(O_2_^-^)	No (23)	Yes (12, 27,28)
		No (23)
Hydroxyl radical		
(HO^-^)	Yes	Yes
	(23,24,27)	(27)
SInglet oxygen	Yes (12,29)	Yes (29)
(O_2_ *)		No (24)
Peroxynitrite	Yes (12,25)	Yes 12,(25)
(ONOO^-^)		
Nitric oxide radical	Yes	Yes (12)
(NO)	(12, 26)	No (26)
Hypochlous acid	Yes (23, 24, 31)	Yes (31)
(HOCL)		
Peroxyl radical	Yes (23)	Yes (23, 30)
(HO_2_..)	No (30)	

ALA/DHLA can also regenerate other antioxidant substances such as vitamin C, vitamin E and the ratio of reduced/oxidized glutathione (GSH/GSSG) [17]. Glutathione is a sulfur tripeptide containing glutamate, cysteine and glycine [32]. Their biosynthesis depend on substrate availability (cysteine), which is enhanced by ALA/DHLA which convert cystine into cysteine and also through gene expression [12]. Considering the latter action, ALA/DHLA is an activator/inducer of translocation of nuclear factor erythroid 2-related factor (Nrf2) to the nucleus for regulation of antioxidant gene expression [33]. GSH which has also a connection with circadian rythms, has many functions over different intracellular processes like ageing, oxidative balance and detoxification of many pollutants [34].

A pro-oxidant effect of ALA is also described in experimental studies, but it is generally observed at higher concentrations than the usual plasmatic concentration observed after oral or intravenous infusion of ALA found in human studies [35, 36]. So far, the pro-oxidant action of ALA/DHLA is not fully understood but could be related to different direct or indirect reactions [37] such as oxidation of DHLA by ubisemiquinone [36], to the maintenance of NrF2 into the cytosol through a linkage to Keap1 protein, resulting in the inhibition of the transcription of cytoprotective genes which include many antioxidant genes [38, 39] or to an ubiquination of Nrf2 in a Keap1-dependent action [38].

## Metal chelator properties

Chelation is a powerful function for most living species. A chelator compound has an important function in most systems because it can counteract agents which have a potential oxidant action. Although chelation therapy plays a prominent role in the clinical treatment of metal intoxication, its use in the treatment of some diseases such as DM, cardiovascular and neurodegenerative entities albeit controversial have been subject to an extensive discussion [40].

ALA/DHLA are considered as chelator compounds because they are able to chelate divalent transient metal ions both *in vivo* and *in vitro* but by different mechanisms of action [41–43]. LA chelates mostly the Mn ^2+^, Cu ^2+,^ Pb ^2+^ and Zn ^2+^. In addition to the previously mentioned ions, DHLA is also able to chelate Hg ^2+^ and Fe ^3+^. Both iron and cupper are recognized as mediators for the production of reactive oxygen species. Moreover, the actions of ALA/DHLA as chelating compounds do not cause metal depletion.

## Action upon transduction signaling systems

### Insulin pathway

ALA has many actions in the insulin metabolic pathways, glucose uptake and glycogen synthesis with some differences between both isomers*. In vitro* studies have shown that R-ALA increases the translocation of GLUT1 and GLUT4 to the plasmatic membrane of adipocytes [21, 41–43] and skeletal muscle cells kept in a culture milieu [42]. Moreover, these events are associated with enhanced activity of proteins of the insulin signaling pathway like insulin receptor (IR), insulin receptor substrate 1 (IRS1), Phosphatidylinositide 3-kinase (PI3K) and protein kinase B (AKT) [42]. These events were also observed when Zucker rats, that are animal models of insulin resistance, were studied [43]. There is still a controversy if this action of ALA is totally insulin-dependent or not [41–43]. Furthermore, in an animal model of isolated rat muscles ALA inhibited glycogen synthesis, and action which is considered to have a pro-oxidant effect [32]. In general the S-ALA did not show a significant effect upon glucose disposal [41–43].

### Nuclear factor kappa B

Nuclear factor kappa B (NFkB) is a transcription factor which is maintained in an inactive form in the cytosol because of its capacity in binding to an inhibitor kinase of NFkB activity, IKK [44]. Oxidative-stress is associated with hyperglycemia [45–48], and the existence of other conditions like viruses, some pathogens, and radiation are supposed to phosphorylate IkB resulting in its degradation, liberation and activation of NFkB which translocates to the nucleus to induce the transcription of several molecules related to inflammation, vascular adhesion and migration of monocytes [44]. ALA inhibits NFkB probably because of its action on the inhibition of the degradation of IkB through modulation of upstream kinases like MAPK [12] or its ability to regenerate vitamin E resulting in inhibition of protein kinase C which is also able to phosphorylate IkB [49]. This action of ALA seems to be independent of its antioxidant action [50]. Several experimental studies *in vitro*[50] and *in vivo* in rats [51] have shown the anti-inflammatory actions of ALA but few studies have addressed this subject in humans [52].

### Adenosine monophosfatase protein kinase

ALA has some important functions in the activity and expression of *5' adenosine monophosphate-activated protein kinase (AMPK)* in peripheral tissues and in brain (hypothalamus). AMPK is considered a multifunctional protein involved in many intracellular pathways related to metabolism, stress response, cell cycle and ageing [53]. Currently, AMPK plays an important role in linking nutritional factors and cancer and is considered to be a promising therapeutic target for cancer prevention and treatment [54].

AMPK is a cellular energy sensor and it is activated by liver kinase B1 (LKB-1) and Ca/calmodulin dependent protein kinase (CaMKK). Activation of AMPK results in down regulation of transcriptional events that promote synthesis of gluconeogenic enzymes, synthesis of fatty acids and up regulation of metabolic pathways resulting in an increased ATP production through glucose and fatty acids oxidation [54]. Moreover, AMPK can induce trasnlocation of GLUT4 to plasma membrane independent of insulin action [54].

So far, it is still unknown which is the mechanism that induces activation of AMPK by ALA in peripheral tissues. According to one study with myoblast cells culture, ALA could increase the activation of AMPK indirectly by activating CaMKK which becomes activated by an increase in intracellular Ca^2+^[55]. The inhibition of CaMKK abolishes this action [55]. Indeed, these actions of ALA-AMPK could also increase energy expenditure by increasing the activity of protein kinase-peroxisome proliferator-activated receptor-gamma coactivator-1alpha (PGC-1-alpha) signaling pathway which is responsible for mitochondrial biogenesis [56]. All the above-mentioned actions resulting from ALA activating AMPK will cause a decrease in plasma glucose, an increase in insulin sensitivity and probably weight loss [53]. Recently it was described an action of ALA upon AMPKK in insulinoma cells culture and isolated islets of Langerhans which resulted in a decreased insulin secretion by 25 to 30% at low (2.8 mmol/L) and high (15 mmmol/L) glucose concentrations [57]. This event was observed both with acute and chronic treatment with ALA. Considering this latter action, another study showed a protective effect of ALA upon 2-deoxy-D-ribose induced oxidative damage and insulin expression in cultured cells and rat islets [58]. This protective action seems to be related to an increased intracellular GSH concentration. Both studies have brought to light important informations about the multiple effects of ALA upon beta cells. The study design, the stimulus and the concentration of ALA used in the experiments could have accounted for the different results that have been found [58].

ALA is also able to modulate the activity of AMPK in the brain by metabolic stresses that inhibit ATP production such as ischemia, hypoxia, glucose deprivation as well as by oxidative stress [59]. Recent studies of experimental neuronal models are pointing to AMPK’s possible roles beyond energy sensing**,***s* ome reporting brain protective effects [60], while others reporting detrimental effects [61]. These discrepancies may be related to the dose of glucose added in the culture medium.

It has also been found that hypothalamic AMPK is important in the central regulation of appetite and energy expenditure and that ALA exerts anti-obesity effects by suppressing hypothalamic AMPK activity [62]. In this setting, ALA has an anorexic property and should be a potential anti-obesity drug [63].

## Relationship between the dose of ALA and its effects in experimental studies

In most experimental studies performed *in vitro* a wide range of ALA doses and glucose concentrations were used, from 10 umol/L to 5 and from 2.0 mmol/L to 15 mmol/L, respectively and a clear dose-dependent response was observed [14, 16, 58–61, 64–66]. The majority of these studies did not give an information about which type of ALA was used: if a racemic admixture of R-ALA and S-ALA, only R-ALA or only S-ALA. In one study a hormonal admixture of ALA was used [65]. The same was noted in animal studies with different purposes [10, 56, 63, 64, 67–72] when the doses used were described as percentage [63] to doses described as mg/rat [68] or described as mg/kg which ranged from 20 to 100 mg/kg [10, 69, 70, 72]. As observed in *in vitro* studies, most of the *in vivo* studies did not give an information about which type of ALA was used. The above-mentioned events could have resulted in protective or deleterious actions of ALA upon different cells as recently pointed out [59]. All these informations must be considered in relation to the absorption rate of the usual prescribed dose of 600 mg which reachs a plasmatic peak of 4 ug/ml, that corresponds to a plasmatic concentration of 0.0194 mmol/L, in about 30 minutes after its ingestion.

## Clinical use of ALA in conditions other than diabetes

### Brain diseases and cognitive dysfunction

Brain is considered to be one of the major glucose and oxygen consumer organs, although it corresponds to only 2% of the total body weight [73]. Neurons and astrocytes are the most active cells of neurometabolism and are considered to be the neurometabolic couple. Currently, oxidative stress, an imbalance between the production of ROS and the antioxidant defenses plays an important function in the occurrence of neurodegenerative diseases as well in brain injuries, mediated mainly by mitochondrial dysfunction [14, 64, 74]. Moreover, the brain has a great sensitivity to oxidative stress-induced damage [75]. Considering ALA as an antioxidant compound its use has been reported in some brain diseases and associated with cognitive dysfunction such as Down syndrome [15] and AD [76, 77].

In a randomized, double blind placebo- controlled study in patients with Down Syndrome with pre-dementia, the use of 900 UI of alpha-tocopherol plus 200 mg of ascorbic acid and 600 mg of ALA for two years did not show improvement in cognitive dysfunction or stabilization of cognitive decline [15]. It is well known that these patients are at an increase risk in developing AD, and oxidative stress is considered to be an important feature of the Down syndrome.

In AD, ALA in association with n-acetylcysteine has shown to have a protective effect upon oxidative stress in fibroblasts decreasing caspase proteins which were responsible for apoptotic processes in patients with AD [14]. In a triple transgenic animal model of AD, ALA was able to improve neurons plasticity and improve many pathways of insulin signaling in the brain similar to the action described with metformin [77, 78]. So far, only two clinical studies have addressed the use of ALA (600 mg/daily) in patients with AD, both studies were open-labeled [79, 80]. One study was conducted with nine patients followed by 12 months [79] and the other with 43 patients followed by 24 months [68], both showing a slowing in the disease progression. Meanwhile, when ALA was associated with exercise training in animal model an increase in some antioxidant enzymes were observed [81].

### Obesity

The increasing prevalence of obesity worldwide is an important epidemiological issue because it is occurring in parallel with the increase in the prevalence of DM and cardiovascular disease (CVD). Moreover, it is well known that both conditions are associated with insulin resistance, an increased plasmatic level of free fatty acids, of pro-inflammatory cytokines such as tumor necrosis factor alfa (TNF-α), interleukin 6 (IL-6) and decreased levels of adiponectin which is considered to be a protective cytokine [65, 82, 83]. The above-mentioned mechanisms seem to be related to oxidative stress and activation of NfKB [84]. ALA has many actions that may result in weight loss such as activation of AMPK in peripheral and brain tissue [59], inhibition of NfKB [44] and adipocyte differentiation [65]. Animal studies showed that rats fed with a high fat diet with ALA supplementation had less weight gain and better plasmatic lipid profile than the control group [65]. Some of these effects, such as the increase in HDL-cholesterol and the decrease in LDL-cholesterol levels were dose dependent. Some studies suggest that the ability of ALA supplementation in preventing insulin resistance might be related in part to the stimulation of AMPK and adiponectin in white adipose tissue [82] and attenuation of monocyte chemokine protein 1 (MCP-1) and TNF-α [71]. The authors suggested that ALA may modulate visceral adipose inflammation.

Data from human studies have shown conflicting results related to lipid metabolism [8, 85–88]. Considering weight loss, the studies have used doses of ALA ranging from 1,000 mg to 1,800 mg for up 20 weeks in obese patients with or without glucose intolerance and have shown a weight loss around 3 kg [8, 87, 88], that corresponds to a 3% of weight loss [87]. It is important to emphasize that the use of sibutramine for one year at a constant dose of 15 mg/daily resulted in a weight loss of about 7% [89]. However, in obese glucose intolerant subjects ALA has only shown an increase in LDL-oxidation in comparison to subjects who completed 12 weeks of ALA used associated with exercise [8]. Another study did not show any advantage of ALA supplementation for two weeks over lipid-induced insulin resistance in obese or overweight subjects [85]. However, an intravenous treatment with 600mg of ALA for two weeks in obese patients with glucose intolerance resulted in improvement of insulin resistance, decreased levels of free fatty acids, LDL-cholesterol as well oxidized LDL, TNF α and IL-6 [86].

The use of 1,200 mg/d of ALA for 10 weeks in an open-label pilot trial in patients with schizophrenia without DM taking atypical antipsychotic drugs showed a weight loss of -2.2 kg ± 2.5 kg. The weight loss was greater in patients taking antihistaminic antipsychotic agents, *mainly clozapine, olanzapine or quetiapine*[88].

Further studies are necessary to address the clinical use of ALA as anti-obesity drug with more complete data about dietary habits including the ingestion of fruits and vegetables which are the main source of antioxidants in a regular diet.

### Nonalcoholic fatty liver disease

Non-alcoholic fatty liver disease (NAFLD) is considered the most prevalent liver disease worldwide. NAFLD is frequently associated with metabolic syndrome, obesity, DM and dyslipidemia [90]. During the natural history of NAFLD up to 25% of the patients developed non-alcoholic steatohepatites (NASH) [91]. Currently, mitochondria dysfunction, oxidative stress and inflammation play a key role in the pathogenesis of NAFLD and NASH [55]. Some animal studies have brought to light the possible mechanisms involved in the action of ALA in NAFLD and NASH in the last years [92, 93]. In one study, the use of ALA was followed by improvement in serum levels of insulin, free fatty acids, glucose, IL-6, triglycerides and markers of inflammation and of innate immune activation (Toll-like receptor 4, TLR4) in liver biopsy [93]. Two other studies using animals fed with a high fat diet showed that ALA induced an increase in uncoupling protein 2 which inhibits electron transport chain resulting in decreased ATP and lipid synthesis [92]. Moreover this action on mitochondria efficiency seems to be related to an increased action of the sirtuin proteins [94]. These proteins have many actions in several intracellular pathways associated with antioxidant defense [95, 96].

### Burning mouth syndrome

Burning mouth syndrome (BMS) is a chronic disease characterized by pain, burning and itching of the oral cavity without association to any systemic disease. Sometimes xerostomia and dysgeusia could be present [97]. BMS is more prevalent in women in the menopause period. In Brazil its prevalence is of 1% [98]. The etiology is probably multifatorial including psychiatric diseases and hypothyroidism [99]. ALA was used during two months in patients with BMS in a dose of 600 mg daily but with conflicting results [98–100].

### Cardiovascular disease and endothelial function

The main cause of mortality in non diabetic as well as in diabetic subjects worldwide is CVD [101]. CVD is multifactorial being the oxidative stress and a pro-inflammatory state considered to be the most important mechanisms involved in the large spectrum of CVD [102]. In this setting, ALA which has antioxidant as well anti-inflammatory actions has been used in several studies, both animal [8–10, 68–70] and human [9, 103] addressing different aspects of CVD.

For instance, the acute use of ALA in a 600 mg dose, associated with 1,000 mg of vitamin C and 600 IU of Vitamin E was able to ameliorate markers of oxidative stress and endothelial dysfunction evaluated by flow-mediated vasodilation (FMD) of the brachial artery in the elderly [7]. The effects of ALA upon endothelial function and markers of oxidative stress were age dependent and it was not observed in young subjects. In contrast, a review of many clinical trials using chronic antioxidant therapy was not able to demonstrate benefits on CVD [103].

In animal models, those fed with a high cholesterol diet, the use of ALA for 12 weeks reduced oxidative stress and weight and improved vascular reactivity [10]. Moreover, a reduction in the wall volume of abdominal aorta with slowing rate of the plaque progression and a reduction of the expression of adhesion molecules in thoracic aorta were also observed. One important finding in this study was the demonstration that ALA decreased the activation of NfKB which regulates the expression of pro-inflammatory genes as well adhesion molecules [10].

The effects and mechanisms of ALA on myocardial infarct size and diabetic cardiomyopathy which is defined as a ventricular dysfunction in diabetic patients without any other cause, were also evaluated in animal studies [66, 67]. Cardiac fibrosis which is the main feature of cardiomyopathy, was investigated in animal with streptozotocin (STZ) DM-induced [66]. In these animals the use of ALA had different actions such as improvement of cardiac function and cardiac fibrosis. Analyzing the left ventricular sections of these animals it was observed a better oxidative stress profile and a decreased expression of transforming growth factor β and smooth muscle actin, both associated with collagen production.

In animal models of ischemia-reperfusion it was demonstrated that ALA ameliorates cardiac dysfunction with a decrease in the infarct size, TNF-α, mieloperoxidase, markers of cell death (lactate dehydrogenase and creatinine kinase), and upregulates gene expression of several antioxidant enzymes [67]. The mechanisms of action of ALA were related to the phosphorylation of PI3K/AKT without any effect on nitric oxide production. Additionally, all these actions were dose dependent, being the dose of 15mg/kg the most effective. No effects with lower or higher doses were observed [67].

A benefit obtained with the use of ALA in hypertension was described and could be related to oxidative stress as well as to the modulation of intracellular Ca^2+^[3]. In animal studies of glucocorticoid-induced hypertension, the use of ALA prevented only dexamethasone induced-hypertension [65]. In human studies the use of ALA as a hypotensive agent presented conflicting results showing improvement or no effect [52, 104]. For instance, the Island Study which used ALA (300 mg/d) plus irbesartan (150 mg/d) for 4 weeks in subjects with metabolic syndrome did not find any difference in blood pressure albeit an improvement in flow mediated vasodilation and a reduction in plasma levels of IL-6 and 8-isoprostane [52]. Moreover it was also demonstrated that both drugs had a synergistic effect upon markers of endothelial dysfunction, inflammation and oxidative stress. It is important to emphasize that this study was not designed to evaluate blood pressure and the dose used of ALA was lower than the doses that are usually employed.

### Cancer

Oxidative stress plays an important role in tumorigenesis [105]. ALA has been used as an anticancer agent mainly in experimental studies of different tumorigenesis cells type with promising results [16, 106–110]. So far the exact molecular mechanisms involved in this action are unknown. Besides its antioxidant acitivity, another possibility could be its relation to the capacity of inducing cellular apoptosis as recently demonstrated in lung cells [106]. This effects may result from activation of caspases proteins induced by endoplasmic reticulum stress [109]. Another hypothesis is associated with the metabolism of cancer cells which convert preferentially glucose to lactate, a mechanism known as the Warburg effect [108]. ALA is the cofactor of pyruvate deydrogenase which converts pyruvate to acetil CoA resulting in a decrease in the formation of lactate [107]. The net effect of this action is the inhibition of glycolysis. Additionally, an inhibition of mTOR (target of rapamycina), a signaling pathway responsible for cell growth and related to insulin receptor phosphorylation- PI3K-AKT activation, has been demonstrated in assays using insulinoma cells [57]. This action resulted in an inhibition of insulin secretion and of beta cells growth [57]. In contrast, recent data have demonstrated an antiapoptotic action of ALA due to a activation of PI3-K/AKT [111]. In addition, in this study it was also showed a direct binding site of ALA to insulin receptor [111]. It is possible to speculate that ALA can act in alternative routes resulting in different effects. The few studies in humans are case reports [111]. In these studies ALA was used associated with other antioxidant agents [111] or with other anticancer drugs [111].

### Miscelaneous disorders

ALA has been used in other clinical conditions such as glaucoma [72] and osteoporosis [112, 113]. Both conditions are associated with an imbalance in the redox state. In a mouse model of glaucoma the increase in intraocular pressure was correlated to increased levels of lipid peroxidation and of oxidative stress-related genes expression in retina. Moreover, in these animals the addition of ALA to the diet enhanced antioxidant defenses, prevented retinal ganglion cell losses without significant intraocular pressure changes.

In a rat model of estrogen deficiency induced by ovariectomy the use of ALA increased bone mineral density (BMD) and decreased inflammatory markers such as TNF- α and IL-6. Besides these effects, the use of ALA also decreased the levels of osteopontin, a protein related to bone resorption [113]. In a model of low BMD induced by high-fat diet, which is a potent inducer of oxidative stress, the ALA supplementation resulted in an increase of the levels of expression of genes related to antioxidant enzymes, BMD, and biomarkers of bone formation, such as osteocalcin, and a down regulation of genes related to bone resorption activity, like osteoprotegerin, in femur biopsy. These studies indicated a possible action of ALA upon maintenance of bone balance.

## Clinical use of ALA in diabetes

DM comprehends a group of genetically and clinically heterogeneous metabolic disorders that are characterized by hyperglycemia which results from a defective insulin secretion and/or activity [114]. The World Health Organization (WHO), estimated that by 2025 there will be 300 million people with DM in the world. DM carries a great risk of morbidity and mortality due to the microvascular and macrovascular complications that can lead to a lower quality of life and life expectancy [115]. Currently, these complications can be postponed by achieving adequate glycemic control, as demonstrated by the Diabetes Control and Complications Trial, the Epidemiology of Diabetes Interventions and Complications and UKPDS [116–118]. However in routine clinical practice good glycemic control is very difficult to be achieved [119, 120]. The aforementioned diabetes-related complications lead to a significant burden to the individual and to the society as a whole [121, 122].

The mechanisms underlying the development of DM related- chronic complications either micro or macrovascular are associated to glycemic control [90, 116–118]. However, many other factors may contribute or have a direct relationship with these complications, such as oxidative stress [123], markers of insulin resistance [124], markers of low-grade inflammation [125], dyslipidemia [126], hypertension [126, 127] and obesity [127]. Indeed, DM-related complications may be considered multifactorial as DM itself [114]. In this context, oxidative stress- related hyperglycemia is considered to be more and more important in the development of DM as well in the development of its related complications [85, 102]. This duet, oxidative stress- related hyperglycemia may induce modifications in signaling pathways responsible for several intracellular processes [102]. Some of these processes are related to inhibition of insulin signaling pathway resulting in insulin resistance [128], reduced insulin gene expression and consequently reduced insulin secretion by beta cells [129]. Moreover, currently there is compelling evidence linking this duet to epigenetic modifications resulting in activation of genetic transcription or repression, silencing the genetic transcription as recently described [45].

In this study it was shown an increasing expression of the subunit p65 of NfKB which resulted in increased transcription of vascular cell adhesion.molecule-1 (VCAM-1) and monocyte chemo attractant molecule-1 (MCP-1) in human aortic endothelial cells under hyperglycemia medium [45]. MCP-1 and VCAM are both related to hyperglycemia-induced arterial pathology. Moreover, this reaction persisted after a long period of normoglycemia establishing the concept of metabolic memory at molecular level.

Recently it was demonstrated also a downregulation of LASY in diabetic animals [18]. In this study either treatment with medium with high glucose or TNF-α resulted in reduction of LASY mRNA [18]. Moreover, a knockout of LAISY showed an intracellular decrease in GSH, superoxide dismutase (SOD) and catalase and an increase in superoxide anion resulting in activation of NfKB, Adding ALA in the cellular medium an up-regulation of LAISY expression was observed [18].

Another important factor in the pathogenesis of diabetes-related complications is the formation of advanced glycation end-products (AGEs) which are derived from intracellular glucose auto-oxidation and non-enzymatic reactions between glucose and intracellular and extracellular proteins [130–133]. AGEs by different mechanisms may damage target cells located in retina, endothelium and glomeruli [131]. AGEs/receptor interactions (AGE/RAGE) may result in altered functions of intra and extracellular proteins, induction of oxidative stress and modifications of matrix proteins resulting in modifications of its functional properties [132]. AGE may also activate PKC which is a signal transduction pathway for regulating many vascular functions like blood flow, permeability, basement membrane thickening and the expression of nitric oxide synthase [133].

Considering the pleiotropic actions of ALA or its reduced form, DHLA in many signaling pathways associated with the pathophysiologic process of DM development as well as the development of its above mentioned chronic-related complications, its use as a therapeutic agent sounds promising.

### Use of ALA in diabetes treatment

ALA has been used in 94 patients with T2M in a randomized, double-blind placebo-controlled study which has tested three antioxidants for three months in association with metformin and glimeperide: ALA, 300 mg/daily, ecosapentanoic acid, 180 mg plus docohexaenoic acid, 120 mg/daily [omega 3 fatty acids] and vitamin E 400 mg/daily [134]. Although an improvement in HbA1c, weight and waist have been observed with ALA, omega 3 fatty acids gave the better results concerning weight loss and glycemic control [134].

Another randomized, placebo-controlled study with 38 patients treated with different doses of ALA (ranging from 300 to 1200 mg/daily), for six months, showed a decrease in HbA1c and fasting blood glucose in the group randomized to ALA [135]. However, there was only a statistical significant difference only with the pooled group of ALA. The reduction of HbA1c was ALA dose-dependent. Moreover, markers of oxidative stress such as lipid peroxidation and oxidative damage of DNA did not show any modification. Another randomized, double blind placebo-controlled study involving 102 patients treated with ALA (600 mg/daily), ALA 600 mg/daily plus 800 mg/daily of α-tocopherol daily for four months showed improvement in some lipid fractions and glucose in the ALA group and ALA plus α-tocopherol but without statistical significance [136]. Data obtained in clinical studies using ALA in the treatment of diabetes-related complications are summarized in Table 2.

**Table 2:** **Clinical studies with ALA in patients with diabetes**

Author	Study type	ALA/other drugs	Analyzed parameters	Total participants	Duration of DM (years)	Follow up (weeks/ years)	Results
***Type 2 diabetes treatment***							
Udupa, AS [134]	Randomized, double- blind placebo-controlled	Vitamin E, omega 3 fatty acids ALA 300 mg All of them daily/orally	Weight, waist glucose	104 with IR	5-10 y	12 w	< HbA1c, weight, waist Better results with omega-3 followed by vitamin E and ALA
Porasuphatana S [135]	Randomized, placebo- controlled	ALA 300 mg -1200 mg/d	HbA1c, FBG	38	2.07 ± 0.26	24 w	<HbA1c, FBG
De Oliveira AM [136]	Randomized, double- blind placebo-controlled	ALA 600 mg or Vitamin E 800 mg or ALA 600 mg plus Vitamin E 800 mgr	HOMA index, glucose, lipid profile insulin	102		16 w	
***Diabetic retinopathy***							
Haritoglou C [139]	Randomized ,double-blind placebo-controlled	ALA 600 mg/daily	Development of macular edema	232 patients with type 2 and 170 with T1D		86 w	no effect
Nebioso M [140]	Randomized not placebo-controlled	ALA 400 mg daily plus vitamins and genistein	ERG	32	NA	4 w	Improvement in ERG
***Diabetic nephropathy***							
Borcea V [143]	Cross-sectional not placebo-controlled	ALA 600 mg/daily/orally	Lipid ROOH, HbA1c, urine albumin, α tocopherol	107 patients [45 with T1D and 29 with T2D]	21.7 ± 11.1 (with ALA); 15.3 ± 10.4 (without ALA)	>12 w	< ROOH < ROOH/(,α tocopherol/cholesterol The decrease was independent of HbA1c and urine albumin level
Cicek M [144]	Randomized not placebo-controlled	ALA 600 mg/ /orally	CIN Plasma creatinine, Cystation C	79	NA	Prior coronary angiography	No effect in the incidence of CIN, creatinine, Cystatin C pré /pos exam
Chang JW [145]	Randomized placebo-controlled	ALA 600 mg/orally	Cholesterol, HbA1c C-reactive protein, oxidizedLDL- ADMA	50 patients on hemodyalisis treatment	NA	12 w	Decrease the level of ADMA
***Diabetes endothelial dysfunction***							
Heinisch BB [147]	Randomized, Controlled,double- blind placebo parallel	Daily 600 mg of ALA IV	Endothelial function endothelium dependent and independent HbA1c, lipid profile	30 patients with TD2	7 ± 6	3 w	Improvement in endothelium dependent function
***Diabetic cardiovascular autonomic neuropathy***							
Pop-Busui R [150]	Prospective, randomized, double- blind, placebo- controlled	ALA 600 mg/twice daily Nicotinamide 750 mg/twice daily Allupurinol 300 mg/daily All of them orally	Autonomic tests PET F_2_ urinary isoprostane HbA1c	44 patients with T1D with mild/moderate cardiovascular autonomic neuropathy and retinopathy or microalbuminuria	27 ± 12	2 y	No improvement in any analyzed parameter
Ziegler D [151]	Randomized, double- blind, placebo- controlled	ALA 800 mg/daily (orally)	Heart rate variability HbA1c Autonomic symptoms	73 patients with T2D	15.3 ± 8.3	16 w	Improvement on root mean square successive difference and power spectrum in low frequency band No difference in the prevalence of symptoms
***Diabetic polyneuropathy***							
ZiegleR D [153] ALADIN I	Randomized,double- blind controlled parallel	ALA:1200 or 600 or 100 mg/daily (orally)	TSS HbA1c	328 patients with T2D with symptomatic peripheral neuropathy	10.4/12.3	3 w	Improvement in TSS HbA1c: no difference
Reljanovic M [154] ALADIN II	Prospective randomized,double- blind controlled	ALA 1200 mg or 600 mg orally	Sensory and motor nerve function	299 patients (T1D and T2D) with symptomatic polyneuropathy	NA	2 y	Improvement in electrophysiological tests HbA1c: no difference
Ziegler D.[155] ALADIN III	Prospective randomized, double- blind controlled	ALA 600 mg/ IV followed by 1800 mg of ALA or placebo orally	TSS NIS	516 patients with T2D with symptomatic polyneuropathy	11	3 w (IV) 24 w (orally)	No effect HbA1c:no difference
Ametov As [157] Sidney I	Randomized, double- blind ,parallel controlled, mono-center	ALA 600 mg IV	TSS	120 (T1D and T2D) with DSPN	15.1 ± 8.8	3	Improvement
Ziegler D [158] Sidney II	Randomized, double- blind, parallel controlled, multicenter	ALA 600 to 1800 mg/orally	TSS	181 (T1D and T2D) with DSPN	14	5	Improvement
Ziegler D [159] Nathan I	Randomized, double- blind, parallel controlled, multicenter	ALA 600 mg/orally	TSS Composite score( NIS–lower limbs plus 7 neurophisiologic tests (NIS-LL+7)	460 (T1D and T2D) with DSPN	13.3	4 years	TSS: no improvement NSI-LL+7:improvement
Ziegler D [160]	Meta-analysis	ALA 600mg IV	TSS, NIS-LL	1258	132 months	3 w	Improvement in TSS (papin-prick,touch-pressure), burning,numbness) Improvement in NIS-LL
Mijnhout GS [161]	Meta-analysis	ALA orally ( 600 to 1800 mg daily) ALA IV (100 to 1200 mg/daily)	TSS	653	NA	3 to 5 w	Improvement in TSS but greater than 30% only in intravenously treated patients

ALA, alpha-lipoic acid; w, weeks; y, years; IV, intravenously; HbA1c, glycated hemoglobin; FBG, fasting blood glucose; HOMA index, homeostasis model assessment; ERG, electroretinogram; ROOH, hidroxiperoxides; CIN, contrast induced nephropathy; ADMA, asymmetric dimethyl-arginine; PET, positron emission tomography; TSS, total symptoms score; NIS, neurophaty impairment score; NIS-LL, neuropathy improvement score of lower limbs; T1D, type 1 diabetes; T2D, type 2 diabetes; IR, insulin resistance; DSPN , distal symmetric sensory-motor polyneuropathy.

### Use of ALA in diabetes-related chronic complications

#### Use of ALA in diabetic retinopathy

ALA has been used to evaluate retinal mitochondria biogenesis in rats in a model of reinstitution of good control after six months of poor metabolic control [137]. In animals without ALA supplementation and under poor glycemic control it was observed a dysregulation of retinal mitochondria biogenesis with a decreased expression of citrase synthase (a marker of mitochondria functional integrity), a decreased number of mitochondria and an increased number of acellular capillaries (a marker of diabetic retinopathy). Moreover, in this study the supplementation of ALA in animals soon after induction of DM prevented most of the above-mentioned alterations [137]. In another experimental study, the treatment with ALA in diabetic animals reduced the markers of oxidative stress, NfKB activation and vascular endothelial growth factor in diabetic retina [138].

A randomized, double-blind placebo-controlled study with 235 patients with T2D and 170 patients with type 1 diabetes (T1D) patients using ALA 600 mg/daily for 2 years (96 weeks) did not show any kind of prevention upon the development of diabetic macular edema [139]. However, using ALA (400 mg/daly) in combination with other anti-oxidants (genistein and vitamins) in 32 diabetic patients an improvement in electroretinogram was observed after 30 days of treatment [140].

#### Use of ALA in diabetic nephropathy

The effects of ALA in the development of diabetic nephropathy was investigated mainly in animal studies. In diabetic animal strepzotocin (STZ)-induced DM and apolipoprotein deficient fed high fat diet the protective effect of ALA supplementation was evaluated in three different time schedule : pre-STZ, simultaneously and pos-STZ. No statistical difference was noted among the groups concerning hyperglycemia, although an attenuation of hyperglycemia was observed in the group pre-STZ. Analyzing the pooled group it was found a reduction in IL-6, urine albumin, urine isoprostane and an increase in erythrocyte GSH in the group under ALA supplementation. The decreased gene expression of superoxide dismutase in diabetic animals was normalized with ALA. Two other animal studies showed interesting data [141, 142]. One study showed that LASY-deficient animals present reduction in antioxidant defense. Moreover, in this study it was also found an overproduction of superoxide in the proximal tubular cells which could be an important event for accelerating the development of diabetic nephropathy [141]. Another study showed opposite action of ALA in animals with STZ-induced DM. In diabetic animals ALA decreased urinary albumin and markers of oxidative stress, but in non-diabetic animals pro-oxidant effects were observed with an increase in urinary albumin, creatinine and markers of oxidative stress [142]. This effect may be at least partially explained by the high dose used in the experiment.

So far, the few human studies which have been done had different objectives [143–145]. One cross-sectional not placebo controlled study using 600 mg/daily of ALA for more than three months in 107 patients with T2D and T1D showed a reduction of lipid hydroxiperoxides. Moreover this reduction was independent of the level of HbA1c and urine albumin [143].

The protective effect of ALA on the development of contrast-induced nephropathy (CIN) was evaluated in 68 patients with DM undergoing coronary angiography. The patients received 600 mg of TA prior to the procedure or no treatment (control group). The two groups had the same incidence of CIN, 8%, without difference in the plasma levels of creatinine, cystatin C pre/pos procedure [144]. The effect of ALA upon asymmetric dimetihylarginine (ADMA) which is an inhibitor of nitric oxid synthase, was investigated in a randomized, control study for 12 week in 50 diabetic patients undergoing hemodyalisis. ALA, 600 mg/daily decreased the levels of ADMA without difference in the other analyzed parameters [145]. An open and non-randomized exploratory study, investigated the effect of 600 mg/daily of ALA for 72 weeks on urinary albumin excretion and plasma levels of trombomodulin in patients with T1D and T2D. A decrease in the level of trombomodulin and no changes in the urinary albumin were observed in the treatment group [146].

#### Use of ALA in endothelial dysfunction

A randomized, controlled, double blind, parallel study with 30 patients with T2D evaluated glycemic control and endothelial responses to intravenous acetylcholine (endothelium dependent) and nitrate (endothelium independent) in order to evaluate the forearm blood flow before and after the use of 600 mg of ALA intravenously for three weeks [147]. A decrease in HbA1c, total cholesterol and triglycerides levels were observed in both groups. However only the patients ALA treated showed an improvement in the endothelium dependent vasodilation. ALA or placebo did not influence endothelium independent vasodilation [147].

#### Use of ALA in diabetic wound healing

An experimental study *in vitro* and *in vivo* has demonstrated a possible benefit of topical application of ALA alone or in combination with other anti-oxidant agents for diabetic wound healing [148]. In this study the expression of RAGE was attenuated in skin wound in diabetic animals when ALA was used in combination with other anti-oxidants agents for one week. Moreover, the use of ALA in combination with other anti-oxidants agents accelerated the skin wound healing with increased expression of vascular endothelial growth factor (VEGF) in the wound area.

#### Use of ALA in diabetic cardiovascular neuropathy

Cardiovascular autonomic neuropathy was evaluated in two human studies. One, randomized, double blind, placebo controlled multicenter study (DEKAN) was conducted in patients with T2D with cardiovascular autonomic neuropathy (CAN) using 800 mg of ALA daily for 16 weeks [149]. Autonomic symptoms and heart rate variability were evaluated before and after the intervention. The intervention with ALA resulted in improvement of some parameters of heart rate variability analysis: root mean square successive difference and power spectrum in low frequency band. No difference was observed in overall symptoms. Another study, a prospective, randomized, double blind, placebo controlled study was performed in 44 patients with T1D presenting any diabetes-related chronic complication (mild non-proliferative retinopathy or microalbuminuria and the presence of cardiac autonomic neuropathy (CAN) defined by an alteration of positron emission tomography (PET) with normal autonomic reflex testing [150]. Patients were submitted to triple anti-oxidant therapy with the objective to target different pathways of oxidative stress damage: inhibition of xantina-oxidase by allopurinol 300 mg/daily, inhibition of oxidative stress by ALA, 600 mg/twice daily and inhibition of poly (ADP-ribose) [11]. In this study no improvement was found in all parameters of autonomic function analysis as well as in urinary levels of isoprostanes, a marker of oxidative stress. Meanwhile a detrimental effect in some regions of left ventricle was observed in PET analysis [150].

#### Use of ALA in diabetic neuropathy

So far, the majority of clinical studies using ALA therapeutically were conducted in order to evaluate its action on diabetic neuropathy. The earlier studies were performed between 1980/1993. In general these studies were uncontrolled, double/single blind or open label, including few patients, had short duration and used a wide range dose either orally or intravenous [11]. The main benefit of ALA was an improvement of symptoms and in distal motor never latencies. It is beyond the scope of this review to analyze each of these studies but they did not have a definite conclusion about the effects of ALA upon diabetic neuropathy. However they have given key information about how to perform other clinical trials better designed to in order to evaluate this topic. It is important to emphasize that at this time the lack of standardization of definition as well as standard criteria for diagnosing diabetic neuropathy are unsolved problems due to different worldwide consensus in the subject. These consensuses have established scores like Neuropathy Symptoms Score (NSS), Total Symptoms Score (TSS), and Neuropathy Impairment Score (NIS) [139, 151, 152] which addressed the intensity and frequency of the most important symptoms of diabetic neuropathy such as pain, burning, numbness and paresthesias. The above-mentioned scores facilitated a normatization of the outcomes in these clinical trials after the 1990’s [153–159]. The first of these studies was the ALADIN (Alpha Lipoic Acid in Diabetic Neuropathy) which was designed to evaluate the efficacy and safety of intravenous ALA during three weeks in three different doses, 1200 mg, 600 mg and 100 mg in comparison to placebo in 328 patients with T2D with symptomatic distal symmetric diabetic polineuropathy (DSPN) [153]. In this study an improvement in TSS was noted in the group using 600 mg vs placebo establishing the safety and efficacy of this dose in comparison to the 1200 mg dose. Moreover, at the dose of 1200 mg a higher rate of adverse events were observed mainly in the gastrointestinal tract. No difference in HbA1c was observed at the end of the study which included 260 patients. This study was followed by ALADIN II which was a long-term trial (24 months) that addressed also electrophysiological tests and Neuropathy Disability Score (NDS) using the same doses of ALA but orally in 299 patients with T2D. In this study only 65 patients could be included in the final analysis because the variability in the electrophysiological tests biased the final results [154]. Although some improvement in sensory nerve function was noted the excessive number of patients excluded should be considered when interpreting these results. The ALADIN III Study has combined 600 mg of ALA intravenously for three weeks followed either by 600 mg of ALA three times daily or placebo for six months (24 weeks) [155]. No improvement in TSS and NIS were observed at the end of the study, although some analyzed parameters such as NIS and TSS presented positive results in short period of ALA intravenously administration (tree weeks). Moreover, a high rate of dropout was noted (about 25%). Recently three randomized, double blind, controlled parallel studies were concluded addressing the efficacy and safety of ALA in diabetic patients with DSPN with TSS or NIS as primary outcome. The majority of the included patients had T2D (up to 70%) [156]. The SYDNEY Trial, a monocenter, short-term study used ALA intravenously during five days a week for three weeks and showed improvement in TSS [157]. The SIDNEY 2 Trial was a multicenter study which used doses of ALA ranging from 600 to 1800 mg daily and also showed an improvement in TSS [158]. Finally, it is important to mention the NATHAN 1 Trial (Neurological Assessment of Thioctic Acid in Diabetic Neuropathy), a multicenter study which used 600 mg of ALA daily for four years with NIS-Lower Limb + seven neurophysiologic tests as primary outcome. In this study after a four-year treatment with ALA in mild-to-moderate DSPN did not influence the primary composite end point but resulted in a significant clinical improvement and prevention of progression of neuropathic impairments. As the primary composite end point did not deteriorate in placebo-treated subjects, secondary prevention of its progression by ALA according to the trial design was not feasible [159]. All these latter studies concluded that the usual dose of 600 mg has efficacy and safety and adverse events, mainly in the gastrointestinal tract, that were dose dependent. Moreover, with one exception (sural latency), all these studies did observe improvement in electrophysiological tests.

Two recent meta-analysis evaluate the use of ALA in diabetic neuropathy [160, 161]. One, included 1,258 diabetic patients treated with 600 mg of ALA, intravenously for three weeks, concluded that individualized TTS such as pain, numbness and burning decreased significantly with ALA in comparison to placebo. Considering the components of NIS-LL an improvement was noted in pin-prick, touch pressure and ankle reflexes [160]. This meta-analysis also pointed out some relevant aspects for conducting future trials to evaluate the benefits of ALA on diabetic neuropathy as follows: homogeneity of the studied patients; duration of the trial; end-points with less variability and finally considering the slowing progression of diabetic neuropathy the end point must have to exclude the latter and address improvement. The other meta-analysis included 653 diabetic patients treated with different doses of ALA oral (two studies) or intravenously (two studies) for three (3 studies) to 5 weeks (one study) concluded that TSS decreased significantly but only in the intravenously study the TSS decrease more than 30% which was considered to be clinically significant [161]. Recently, a non-randomized, open-label and prospective study has shown an improvement in pain and eletroneurographic parameters, mainly in sensory nerve conduction, in 50 patients with diabetes and symmetric sensorimotor polyneuropathy treated with a new oral formulation combining ALA 400 mg/daily and superoxide dismutase 140 IU/daily for four months [162].

*Recently, a randomized, open label, parallel study showed no benefit of adding methylcobalamine(750 ug) and ALA(100 mg) to pregabalin (75 mg) for 12 weeks in parameters of nerve function and pain evaluation*[163]*. Another prospective, observational study showed that after 21 months patients using pregabalin had better improvement in symptoms of diabetic neuropathy in comparison to patients using either carbamazepine and ALA*[164]*.*

## Adverse events

Adverse events related to the administration of ALA were described mainly in clinical trials [153, 158, 159] but generally without difference when compared with placebo. The majority of these adverse events were dose-dependent and in the gastrointestinal tract (nausea, vomiting, dyspepsia and abdominal pain). However other events were also described like pruritus, bronchitis and skin ulceration. Recently it was described a case of insulin autoimmune syndrome probably associated with the use of ALA as a nutritional supplement [165].

## Conclusions

ALA a natural anti-oxidant is a cofactor of mitochondrial enzymes of oxidative metabolism like pyruvate dehydrogenase which link glycolysis to citric acid cycle, and α-keto-glutarate dehydrogenase. ALA and its reduced form DHLA have many biological functions in different intracellular systems resulting in a wide range of actions such as antioxidant protection, chelation of metal ions, regeneration of other antioxidant agents such as vitamin C, E and glutathione. Moreover, ALA/DHLA can also act in multiple signaling transduction pathways, like insulin, nuclear factor kappa B (NFkB), nitric oxide synthesis and cellular apoptosis. Also, ALA/DHLA can modulate directly or indirectly the expression of protein kinase C and AMPK that are both key enzymes in many downstream systems. All the aforementioned described actions confer some properties to ALA/DHLA that are not just related to its reactive oxygen and nitrogen species scavenger but also to glucose disposal, inflammation, tumorigenesis, endothelial function, appetite regulation and cognitive function. To date, the majority of these actions have been addressed mainly in experimental studies which used a wide range dose of ALA *in vitro* as well as *in vivo*. We can also consider that for instance, the used dose was greater than the physiological dose reached with the usual clinically used oral dose of ALA. It is also to be mentioned that in most of these studies it was not well defined which type of ALA has been used. Finally, the translation of all these pooled experimental data to human studies is a subject for further research.

## Perspectives

Currently, there are compelling evidences linking oxidative damage to the majority of chronic diseases with increasing prevalence worldwide such as obesity, DM, CVD and AD. Considering the pleiotropic action of ALA upon different pathways associated with the above mentioned diseases, its use as a potential therapeutical agent seems promising. So far, although in a limited number, the majority of clinical studies, performed in randomized double-blind and placebo-controlled ways, have been done in diabetic patients with DSPN. Future clinical studies, also randomized double-blind and placebo-controlled with adequate sample calculation, homogeneity of the studied patients, longer duration and a minimal variability in the established outcomes are needed in order to asses the benefit of ALA upon other diabetes-related chronic complications. Considering the latter statement it will be an important issue to define the use of ALA as primary or secondary therapeutic intervention. Also, the same aforementioned type of studies with the same criteria must be addressed in other clinical conditions such as obesity, CVD and AD. Another important question to be answered by these clinical studies is when we are going to start its use according to the natural evolution of each disease in order to reach a benefit. We need also more experimental studies to evaluate and define if the pro-oxidant action of ALA is dose-dependent. These studies may also give us more information about the use of lipoic acid synthase as a molecular target for increasing the mitochondrial levels of ALA. Another point to be addressed in these studies is the possibility that hyperglycemia can affect different pathways resulting in a toxicity which could be independent of oxidative stress as recently discussed [166]. The role of endoplasmic reticulum stress has been pointed out as an important mechanism leading to diabetes-related complications which is independent of oxidative stress.

Finally, although our review had the objective to extended our clinical and biological knowledge about ALA we still need more information about this multifunctional compound to spread its use in routine clinical practice.

## Authors’ original submitted files for images

Below are the links to the authors’ original submitted files for images. Authors’ original file for figure 1 Authors’ original file for figure 2

## Abbreviations

ALA: Alpha-lipoic acid
TA: Thioctic acid
DHLA: Dihydrolipoic acid
GSH: Glutathione peroxidase
NFkB: Nuclear factor kappa B
DM: Diabetes mellitus
acetyl-CoA: Acetyl coenzyme A
Nrf2: Nuclear factor erythroid 2-related factor
IR: Insulin receptor
IRS1: Insulin receptor substrate 1
PI3K: Phosphatidylinositide 3-kinase
AKT: Protein kinase B
IkB: Inhibitors of nuclear factor kappa B
AMPK: 5' adenosine monophosphate-activated protein kinase
LKB-1: Liver kinase B1
CaMKK: Ca/calmodulin dependent protein kinase
PGC-1-alpha: Protein kinase-peroxisome proliferator-activated receptor-gamma coactivator-1alpha
CVD: Cardiovascular disease
TNF-α: Tumor necrosis factor alpha
IL-6: Interleukine 6
MCP-1: Monocyte chemokine protein-1
NAFLD: Non-alcoholic fatty liver disease
NASH: Non-alcoholic steatohepatites
BMS: Burning mouth syndrome
TLR4: Toll-like receptor 4
FMD: Flow-mediated vasodilation
STZ: Streptozotocin
BMD: Bone mineral density
T2D: Type 2 diabetes
WHO: World Health Organization
VCAM-1: Vascular cell adhesion.molecule-1
AGEs: Advanced glycation end-products
AGE/RAGE: AGEs/receptor interactions
LASY: Lipoic acid synthase
T1D: Type 1 diabetes
CIN: Contrast-induced nephropathy
ADMA: Asymmetric dimetihylarginine
VEGF: Vascular endothelial growth factor
CAN: Cardiac autonomic neuropathy
PET: Positron emission tomography
NSS: Neuropathy symptoms score
TSS: Total symptoms score
NIS: Neuropathy impairment score
ALADIN: Alpha lipoic acid in diabetic neuropathy
DSPN: Symptomatic distal symetric diabetic polineuropathy
NDS: Neuropathy disability score
NATHAN: Neurological assessment of thioctic acid in diabetic neuropathy.

## References

[1] Golbidi S, Badran M, Laher I (2011). Diabetes and alpha lipoic Acid. Front Pharmacol.

[2] Reed LJ (1998). From lipoic acid to multi-enzyme complexes. Protein Sci.

[3] Shay KP, Moreau RF, Smith EJ, Smith AR, Hagen TM (2009). Alpha-lipoic acid as a dietary supplement: molecular mechanisms and therapeutic potential. Biochim Biophys Acta.

[4] Snell EE, Strong FM, Peterson WH (1937). **Growth factor for bacteria.VI Fractionation and properties of an accessory factor for lactic acid** bacteria. Bichem J.

[5] Reed LJ, De Busk BG, Gunsalus IC, Hornberger CS (1951). Cristalline alpha-lipoic acid: a catalytic agent associated with pyruvate dehydrogenase. Science.

[6] Bock E, Schneeweiss J (1959). Ein Beitrag zur Therapie der neuropathia diabetic. Munchner Med Wochenschrift.

[7] Wray DW, Nishyyama SK, Harris RA, Zhao J, McDaniel J, Fjeldstad AS, Witman MA, Ives SJ, Barrett-O’Keefe Z, Richardson RS (2012). Acute reversal of endothelial dysfunction in the elderly following antioxidant consumption. Hypertension.

[8] McNeilly AM, Davison GW, Murphy MH, Nadeem N, Trinick T, Duly E, Novials A, McEneny J (2011). Effect of α-lipoic acid and exercise training on cardiovascular disease risk in obesity with impaired glucose tolerance. Lipids Health Dis.

[9] Zhang WJ, Bird KE, McMillen TS, LeBoeuf RC, Hagen TM, Frei B (2008). Dietary alpha-lipoic acid supplementation inhibits atherosclerotic lesion development in apolipoprotein E-deficient and apolipoprotein E/low-density lipoprotein receptor-deficient mice. Circulation.

[10] Ying Z, Kherada N, Farrar B, Kampfrath T, Chung Y, Simonetti O, Deiuliis J, Desikan R, Khan B, Villamena F, Sun Q, Parthasarathy S, Rajagopalan S (2010). Lipoic acid effects on established atherosclerosis. Life Sci.

[11] Ziegler D, Reljanovic M, Mehnert H, Gries FA (1999). α Lipoic acid in the treatment of diabetic polyneuropathy in Germany: current evidence from clinical trials. Exp Clin Endocrinol Diabetes.

[12] Packer L, Kraemer K, Rimbach G (2001). Molecular aspects of lipoic acid in the prevention of diabetes complications. Nutrition.

[13] Vasdev S, Ford CA, Parai S, Longerich L, Gadag V (2000). Dietary alpha-lipoic acid supplementation lowers blood pressure in spontaneously hypertensive rats. J Hypertens.

[14] Moreira PI, Harris PLR, Zhu X, Santos MS, Oliveira CR, Smith MA, Perry G (2007). Lipoic acidi and n-acetyl cysteine decrease mitochondrial-related oxidative stress in Alzheimer disease patient fibroblasts. J Alzheimers Dis.

[15] Lott IT, Doran E, Nguyen VQ, Tournay A, Head E, Gillen DL (2011). Down syndrome and dementia: a randomized, controlled trial of antioxidant supplementation. Am J Med Genet A.

[16] Al Abdan M (2012). Alfa-lipoic acid controls tumor growth and modulates hepatic redox state in Ehrlich-ascites-carcinoma-bearing mice. Scientific World Journal.

[17] Szelag M, Mikulski D, Molski M (2012). Quantum-chemical investigation of the structure and the antioxidant properties of α-lipoic acid and its metabolites. J Mol Model.

[18] Padmalayam I, Hasham S, Saxena U, Pillarisetti S (2009). **Lipoic acid** synthase (LASY): a novel role in inflammation, mitochondrial function, and insulin resistance. Diabetes.

[19] McLain AL, Cormier PJ, Kinter M, Szweda LI (2013). Glutathionylation of α-ketoglutarate dehydrogenase: the chemical nature and relative susceptibility of the cofactor lipoic acid to modification. Free Radic Biol Med.

[20] Hassan BH, Cronan JE (2011). Protein-protein interactions in assembly of lipoic acid on the 2-oxoacid dehydrogenases of aerobic metabolism. J Biol Chem.

[21] Moini H, Tirosh O, Park YC, Cho KJ, Packer L (2002). R-alpha-lipoic acid action on cell redox status, the insulin receptor, and glucose uptake in 3T3-L1 adipocytes. Arch Biochem Biophys.

[22] A-Vadlapudi AD, Vadlapatla RK, Mitra AK (2012). Sodium dependent multivitamin transporter (SMVT): a potential target for drug delivery. Curr Drug Targets.

[23] Scott BC, Aruoma OI, Evans PJ, O’Neill C, Van der Vliet A, Cross CE, Tritschler H, Halliwell B (1994). Lipoic and dihydrolipoic acids as antioxidants. A critical evaluation. Free Radic Res.

[24] Packer L, Witt EH, Tritschler HJ (1995). Alpha-lipoic acid as a biological antioxidant. Free Rad Biol Med.

[25] Trujillo M, Radi R (2002). Peroxynitrite reaction with the reduced and the oxidized forms of lipoic acid: new insights into the reaction of peroxynitrite with thiols. Arch of Biochem and Biophys.

[26] Vriesman MF, Haenen GR, Westerveld GJ, Paquay JB, Voss HP, Bast A (1997). A method for measuring nitric oxide radical scavenging activity. Scavenging properties of sulfur-containing compounds. Pharm World Sci.

[27] Suzuki YJ, Tsuchiya M, Packer L (1991). Thioctic acid and dihydrolipoic acid are novel antioxidants which interact with reactive oxygen species. Free Radic Res Commun.

[28] Suzuki YJ, Tsuchiya M, Packer L (1993). Antioxidant activities of dihydrolipoic acid and its structural homologues. Free Radic Res Commun.

[29] Devasagayam TP, Di Mascio P, Kaiser S, Sies H (1991). Singlet oxygen induced single-strand breaks in plasmid pBR322 DNA: the enhancing effect of thiols. Biochim Biophys Acta.

[30] Kagan VE, Shvedova A, Serbinova E, Khan S, Swanson C, Powell R, Packer L (1992). Dihydrolipoic acid–a universal antioxidant both in the membrane and in the aqueous phase. Reduction of peroxyl, ascorbyl and chromanoxyl radicals. Biochem Pharmacol.

[31] Haenen GR, Bast A (1991). Scavenging of hypochlorous acid by lipoic acid. Biochem Pharmacol.

[32] Newsholme P, Rebelato E, Abdulkader F, Krause M, Carpinelli A, Curi R (2012). Reactive oxygen and nitrogen species generation, antioxidant defenses, and β-cell function: a critical role for amino acids. J Endocrinol.

[33] Koriyama Y, Nakayama Y, Matsugo S, Kato S (2013). Protective effect of lipoic acid against oxidative stress is mediated by Keap1/Nrf2-dependent heme oxygenase-1 induction in the RGC-5 cellline. Brain Res.

[34] Wilking M, Ndiaye M, Mukhtar H, Ahmad N (2013). Circadian rythms connections to oxidative stress: implications for human health. Antioxid Redox Signal.

[35] Dicter N, Madar Z, Tirosh O (2002). Alpha-lipoic acid inhibits glycogen synthesis in rat soleus muscle via its oxidative activity and the uncoupling of mitochondria. J Nutr.

[36] Rouchette L, Ghibu S, Richard C, Zeller M, Cottin Y, Vergely C (2013). Direct and indirect antioxidant properties of α -lipoic acid. Mol Nutr Food Res.

[37] Moini H, Packer L, Saris N-E (2002). Antioxidant and prooxidant activities of α-lipoic acid and dihydrolipoic acid. Toxicol Appl Pharmacol.

[38] Zhang DD, Lo S-C, Cross JV, Templeton DJ, Hannink M (2004). keap1 is a redox-regulated substrate adaptor protein for a Cul3-dependent ubiquitin ligase complex. Mol Cell Biol.

[39] Dinkova-kostova AT, Talalay P (2008). Direct and indirect antioxidant properties of inducers of cytoprotective proteins. Mol Nutr Food Res.

[40] Frizzell N, Baynes JW (2013). Chelation therapy: overlooked in the treatment and prevention of diabetes complications?. Future Med Chem.

[41] Ou P, Tritscheler HJ, Wolff SP (1995). Thioctic (lipoic acid): a therapeutical metal-chelating antioxidant?. Biochem Pharmacol.

[42] Bast A, Haenen GR (2003). Lipoic acid: a multifunctional antioxidant. Biofactors.

[43] Smith AR, Shenvi SV, Widlansky M, Suh JH, Hagen TM (2004). Lipoic acid as a potential therapy for chronic diseases associated with oxidative stress. Curr Med Chem.

[44] Yaworsky K, Somwar R, Ramlal T, Tritschler HJ, Klip A (2000). Engagement of the insulin-sensitive pathway in the stimulation of glucose transport by alpha-lipoic acid in 3T3-L1 adipocytes. Diabetologia.

[45] Estrada DE, Ewart HS, Tsakiridis T, Volchuk A, Ramlal T, Tritschler H, Klip A (1996). Stimulation of glucose uptake by the natural coenzyme alpha-lipoic acid/thioctic acid: participation of elements of the insulin signaling pathway. Diabetes.

[46] Henriksen EJ, Jacob S, Streeper RS, Fogt DL, Hokama JY, Tritschler HJ (1997). Stimulation by alpha-lipoic acid of glucose transport activity in skeletal muscle of lean and obese Zucker rats. Life Sci.

[47] Yamamoto Y, Gaynor RB (2001). Therapeutical potential of inhibition of the NFkb pathway in the treatment of inflammation and cancer. J Clin Invest.

[48] El-Osta A, Brasacchio D, Yao D, Pocai A, Jones PL, Roeder RG, Cooper ME, Brownlee M (2008). Transient high glucose causes persistent epigenetic changes and altered gene expression during subsequent normoglycemia. J Exp Med.

[49] Bierhaus A, Chevion S, Chevion M, Hofmann M, Quehenberger P, Illmer T, Luther T, Berentshtein E, Tritschler H, Müller M, Wahl P, Ziegler R, Nawroth PP (1997). Advanced glycation end product-induced activation of NF-kappaB is suppressed by alpha-lipoic acid in cultured endothelial cells. Diabetes.

[50] Ying Z, Kampfrath T, Sun Q, Parthasarathy S, Rajagopalan S (2011). Evidence that α-lipoic acid inhibits NF-κB activation independent of its antioxidant function. Inflamm Res.

[51] Zembron-Lacny A, Gajeswski M, Naczac M, Dziewiecka H, Siatkkowski I (2013). Physical activity and alpha-lipoic acid modulate inflammatory response through changes in thiol redox status. J Physiolo.

[52] Sola S, Mir MQ, Cheema FA, Khan-Merchant N, Menon RG, Parthasarathy S, Khan BV (2005). Irbesartan and lipoic acid improve endothelial function and reduce markers of inflammation in the metabolic syndrome: results of the Irbesartan and Lipoic Acid in Endothelial Dysfunction (ISLAND) study. Circulation.

[53] Steinberg GR, Kemp BE (2009). AMPK in health and disease. Physiol Rev.

[54] Zhou G, Myers R, Li Y, Chen Y, Shen X, Fenyk-Melody J, Wu M, Ventre J, Doebber T, Fujii N, Musi N, Hirshman MF, Goodyear LJ, Moller DE (2001). Role of AMP-activated protein kinase in mechanism of metformin action. J Clin Invest.

[55] Shen QW, Zhu MJ, Tong J, Ren J, Du M (2007). Ca2+/calmodulin-dependent protein kinase kinase is involved in AMP-activated protein kinase activation by alpha-lipoic acid in C2C12 myotubes. Am J Physiol Cell Physiol.

[56] Wang Y, Li X, Guo Y, Chan L, Guan X (2010). Alpha-Lipoic acid increases energy expenditure by enhancing adenosine monophosphate-activated protein kinase-peroxisome proliferator-activated receptor-gamma coactivator-1alpha signaling in the skeletal muscle of aged mice. Metabolism.

[57] Targonsky ED, Dai F, Koshkin V, Karaman GT, Gyulkhandanyan AV, Zhang Y, Chan CB, Wheeler MB (2006). Alpha-lipoic acid regulates AMP-activated protein kinase and inhibits insulin secretion from beta cells. Diabetologia.

[58] Koh G, Yang EJ, Kim MK, Lee SA, Lee DH (2013). Alpha-lipoic acid treatment reverses 2-deoxy-D-ribose-induced oxidative damage and suppression of insulin expression in pancreatic β-cells. Biol Pharm Bull.

[59] Ramamurthy S, Ronnet G (2012). AMP-activated protein kinase (AMPK) and energy sensing in the brain. Exp Neurobiol.

[60] Blazquez C, Geelen MJ, Velasco G, Guzmán M (2001). The AMP activated protein kinase prevents ceramide synthesis de novo and astrocytes. FEBS Lett.

[61] Nakatsu Y, Kotake Y, Hino A, Ohta S (2008). Activation of AMO-activated protein kinase by tributyltin induces neuronal cell death. Toxicol Appl Pharmacol.

[62] Kim MS, Park JY, Namkoong C, Jang PG, Ryu JW, Song HS, Yun JY, Namgoong IS, Ha J, Park IS, Lee IK, Viollet B, Youn JH, Lee HK, Lee KU (2004). Anti-obesity effects of alpha-lipoic acid mediated by suppression of hypothalamic AMP-activated protein kinase. Nat Med.

[63] Seo EY, Ha AW, Kim WK (2012). α lipoic acid reduced weight gain and improved lipid profile in rats fed with high fat diet. Nutr Res Pract.

[64] Tomassoni D, Amenta F, Amantini C, Farfariello V, Di Cesare ML, Nwankwo IE, Marini C, Tayebati SK (2013). Brain activity of thioctic acid enantiomers: in vitro and in vivo studies in an animal model of cerebrovascular injury. Int J Mol Sci.

[65] Cho KJ, Moon HE, Moini H, Packer L, Yoon DY, Chung AS (2003). Alpha-lipoic acid inhibits adipocyte differentiation by regulating pro-adipogenic transcription factors via mitogen-activated protein kinase pathways. J Biol Chem.

[66] Wang Y, Dong W, Ding X, Wang F, Wang Y, Chen X, Yu L, Li X, Zhang A, Peng Y (2012). Protective effect of α-lipoic acid on islet cells co-cultured with 3T3L1 adipocytes. Exp Ther Med.

[67] Tian YF, He CT, Chen YT, Hsieh PS (2013). Lipoic acid suppresses portal endotoxemia-induced steatohepatitis and pancreatic inflammation in rats. World J Gastroenterol.

[68] Ong SL, Vohra H, Zhang Y, Sutton M, Whitworth JA (2013). The effect of alpha-lipoic acid on mitochondrial superoxide and glucocorticoid-induced hypertension. Oxid Med Cell Longev.

[69] Li CJ, Lv L, Li H, Yu D (2012). Cardiac fibrosis and dysfunctionin experimental diabetic cardiomyopathy are amelioreted by alpha-lipoic acid. Cardiovasc Diabetol.

[70] Deng C, Sun Z, Tong G, Yi W, Ma L, Zhao B, Cheng L, Zhang J, Cao F, Yi D (2013). α-Lipoic acid reduces infarct size and preserves cardiac function in rat myocardial ischemia/reperfusion injury through activation of PI3K/Akt/Nrf2 pathway. PLoS One.

[71] Yi X, Nickeleit V, James LR, Maeda N (2011). α-Lipoic acid protects diabetic apolipoprotein E-deficient mice from nephropathy. J Diabetes Complications.

[72] Inman DM, Lambert WS, Calkins DJ, Horner PJ (2013). α-Lipoic acid antioxidant treatment limits glaucoma-related retinal ganglion cell death and dysfunction. PLoS One.

[73] Jha MK, Jeon S, Suk K (2012). Pyruvate Dehydrogenase Kinases in the nervous system: their principal functions in Neuronal-glial metabolic interaction and Neuro-metabolic disorders. Curr Neuropharmacol.

[74] Carvalho C, Cardoso S, Correia SC, Santos RX, Santos MS, Baldeiras I, Oliveira CR, Moreira PI (2012). Metabolic alterations induced by sucrose intake and Alzheimer’s disease promote similar brain mitochondrial abnormalities. Diabetes.

[75] Maher PA, Schubert DR (2009). Metabolic links between diabetes and Alzheimer’s disease. Expert Rev Neurother.

[76] Piau A, Nourhashémi F, Hein C, Caillaud C, Vellas B (2011). Progress in the development of new drugs in Alzheimer’s disease. J Nutr Health Aging.

[77] Sancheti H, Akopian G, Yin F, Brinton RD, Walsh JP, Cadenas E (2013). Age-dependent modulation of synaptic plasticity and insulin mimetic effect of lipoic acid on a mouse model of Alzheimer’s disease. PLoS One.

[78] Gupta A, Bisht B, Dey CS (2011). Peripheral insulin-sensitizer drug metformin ameliorates neuronal insulin resistance and Alzheimer’s-like changes. Neuropharmacology.

[79] Maczurek A, Hager K, Kenklies M, Sharman M, Martins R, Engel J, Carlson DA, Münch G (2008). Lipoic acid as an anti-inflammatory and neuroprotective treatment for Alzheimer’s disease. Adv Drug Deliv Rev.

[80] Hager K, Kenklies M, McAfoose J, Engel J, Münch G (2007). Alpha-lipoic acid as a new treatment option for Alzheimer’s disease–a 48 months follow-up analysis. J Neural Transm Suppl.

[81] Cho JY, Um HS, Kang EB, Cho IH, Kim CH, Cho JS, Hwang DY (2010). The combination of exercise training and alpha-lipoic acid treatment has therapeutic effects on the pathogenic phenotypes of Alzheimer’s disease in NSE/APPsw-transgenic mice. Int J Mol Med.

[82] Prieto-Hontoria PL, Pérez-Matute P, Fernández-Galilea M, Alfredo Martínez J, Moreno-Aliaga MJ (2013). Effects of lipoic acid on AMPK and adiponectin in adipose tissue of low- and high-fat-fed rats. Eur J Nutr.

[83] Deiuliis JA, Kampfrath T, Ying Z, Maiseyeu A, Rajagopalan S (2011). Lipoic acid attenuates innate immune infiltration and activation in the visceral adipose tissue of obese insulin resistant mice. Lipids.

[84] Lamb RE, Goldstein BJ (2008). Modulating an oxidative-inflmmatory cascade: potential new treatment strategy for improving glucose metabolism, insulin resistance, and vascular function. Int J Clin Pract.

[85] Xiao C, Giacca A, Lewis GF (2011). Short-term oral α-lipoic acid does not prevent lipid-induced dysregulation of glucose homeostasis in obese and overweight nondiabetic men. Am J Physiol Endocrinol Metab.

[86] Zhang Y, Han P, Wu N, He B, Lu Y, Li S, Liu Y, Zhao S, Liu L, Li Y (2011). Amelioration of lipid abnormalities by α-lipoic acid through antioxidative and anti-inflammatory effects. Obesity (Silver Spring).

[87] Koh EH, Lee WJ, Lee SA, Kim EH, Cho EH, Jeong E, Kim DW, Kim MS, Park JY, Park KG, Lee HJ, Lee IK, Lim S, Jang HC, Lee KH, Lee KU (2011). Effects of alpha-lipoic acid on body weight in obese subjects. Am J Med.

[88] Ratliff JC, Palmese LB, Reutenauer EL, Tek C (2013). An open-label pilot trial of alpha-lipoic acid for weight loss in patients with schizophrenia without diabetes. Clin Schizophr Relat Psychoses.

[89] Lean MEJ (1997). Sibutramine: a review of clinical efficacy. In J Obes.

[90] Lazo M, Clark JM (2008). The epidemiology of nonalcoolic faty liver disease: a global perspective. Semin Liver Dis.

[91] Dixon JB, Bhathal PS, O’Brien PE (2001). Nonalcoholic fatty liver disease: predictors of nonalcoholic steatohepatitis and liver fibrosis in the severely obese. Gastroenterology.

[92] Valdecantos MP, Pérez-Matute P, González-Muniesa P, Prieto-Hontoria PL, Moreno-Aliaga MJ, Martínez JA (2012). Lipoic acid administration prevents nonalcoholic steatosis linked to long-term high-fat feeding by modulating mitochondrial function. J Nutr Biochem.

[93] Jung TS, Kim SK, Shin HJ, Jeon BT, Hahm JR, Roh GS (2012). α-lipoic acid prevents non-alcoholic fatty liver disease in OLETF rats. Liver Int.

[94] Valdecantos MP, Pérez-Matute P, González-Muniesa P, Prieto-Hontoria PL, Moreno-Aliaga MJ, Martínez JA (2012). Lipoic acid improves mitochondrial function in nonalcoholic steatosis through the stimulation of sirtuin 1 and sirtuin 3. Obesity.

[95] Chen WL, Kang CH, Wang SG, Lee HM (2012). α-Lipoic acid regulates lipid metabolism through induction of sirtuin 1 (SIRT1) and activation of AMP-activated protein kinase. Diabetologia.

[96] Chong ZZ, Shang YC, Wang S, Maiesse K (2012). SIRT1: new avenues of discovery for disorders of oxidative stress. Expert Opin Ther Targets.

[97] Gurvits GE, Tan A (2013). Burning mouth syndrome. World J Gastroenterol.

[98] Cavalcanti DR, da Silveira FR (2009). Alpha lipoic acid in burning mouth syndrome–a randomized double-blind placebo-controlled trial. J Oral Pathol Med.

[99] Femiano F, Lanza A, Buonaiuto C, Gombos F, Nunziata M, Cuccurullo L, Cirillo N (2008). Burning mouth syndrome and burning mouth in hypothyroidism: proposal for a diagnostic and therapeutic protocol. Oral Surg Oral Med Oral Pathol Oral Radiol Endod.

[100] Femiano F (2002). Burning mouth syndrome (BMS): an open trial of comparative efficacy of alpha-lipoic acid (thioctic acid) with other therapies. Minerva Stomatol.

[101] Hu G, Jousilhati P, Qiao Q, Katoh S (2005). Sex differences in cardiovascular and total mortality among diabetic and non-diabetic individuals with or without history of myocardial infarction. Diabetologia.

[102] Brownlee M (2001). Biochemistry and molecular cell biology of diabetic complications. Nature.

[103] Kris-Etherton PM, Lichtenstein AH, Howard BV, Steinberg D, Witztum JL, Nutrition Committee of the American Heart Association Council on Nutrition, Physical Activity, and Metabolism (2004). Antioxidant vitamin supplements and cardiovascular disease. Circulation.

[104] McMackin CJ, Widlansky ME, Hamburg NM, Huang AL, Weller S, Holbrook M, Gokce N, Hagen TM, Keaney JF, Vita JA (2007). Effect of combined treatment with alpha-Lipoic acid and acetyl-L-carnitine on vascular function and blood pressure in patients with coronary artery disease. J Clin Hypertens (Greenwich).

[105] Durand M, Mach N (2013). Alpha lipoic acid and its antioxidant against cancer and diseases of central sensitization. Nutr Hosp.

[106] Michikoshi H, Nakamura T, Sakai K, Suzuki Y, Adachi E, Matsugo S, Matsumoto K (2013). α-Lipoic acid-induced inhibition of proliferation and met phosphorylation in human non-small cell lung cancer cells. Cancer Lett.

[107] Feuerecker B, Pirsig S, Seidl C, Aichler M, Feuchtinger A, Bruchelt G, Senekowitsch-Schmidtke R (2012). Lipoic acid inhibits cell proliferation of tumor cells in vitro and in vivo. Cancer Biol Ther.

[108] Kim JI, Cho SR, Lee CM, Park ES, Kim KN, Kim HC, Lee HY (2012). Induction of ER stress-mediated apoptosis by α-Lipoic Acid in A549 cell lines. Korean J Thorac Cardiovasc Surg.

[109] Mantovani G, Macciò A, Madeddu C, Mura L, Gramignano G, Lusso MR, Murgia V, Camboni P, Ferreli L (2003). The impact of different antioxidant agents alone or in combination on reactive oxygen species, antioxidant enzymes and cytokines in a series of advanced cancer patients at different sites: correlation with disease progression. Free Radic Res.

[110] Guais A, Baronzio G, Sanders E, Campion F, Mainini C, Fiorentini G, Montagnani F, Behzadi M, Schwartz L, Abolhassani M (2012). Adding a combination of hydroxycitrate and lipoic acid (METABLOC™) to chemotherapy improves effectiveness against tumor development: experimental results and case report. Invest New Drugs.

[111] Diesel B, Kulhanek-Heinze S, Höltje M, Brandt B, Höltje HD, Vollmar AM, Kiemer AK (2007). Alpha-lipoic acid as a directly binding activator of the insulin receptor: protection from hepatocyte apoptosis. Biochemistry.

[112] Polat B, Halici Z, Cadirci E, Albayrak A, Karakus E, Bayir Y, Bilen H, Sahin A, Yuksel TN (2013). The effect of alpha-lipoic acid in ovariectomy and inflammation-mediated osteoporosis on the skeletal status of rat bone. Eur J Pharmacol.

[113] Xiao Y, Cui J, Shi Y, Le G (2011). Lipoic acid increases the expression of genes involved in bone formation in mice fed a high-fat diet. Nutr Res.

[114] Skyler J, Oddo C (2002). Diabetes trends in the USA. Diabetes Metab Res Ver.

[115] Wild S, Roglic G, Green A, Sicree R, King H (2004). Global prevalences of diabetes, estimates for the year 2000 and projections for 2030. Diabetes Care.

[116] The Diabetes Control and Complications Trial Study Research Group (1993). The effect of intensive treatment of diabetes on the development and progression of long-term complications in insulin-dependent diabetes mellitus. N Eng J Med.

[117] The Diabetes Control and Complications Trial/Epidemiology of Diabetes Interventions and Complications (DCCT/EDIC) Study Research Group (2005). Intensive diabetes treatment and cardiovascular disease in patients with diabetes type 1. N Engl J Med.

[118] Stratton IM, Adler AI, Neil AW, Matthews DR, Manley SE, Cull CA, Hadden D, Turner RC, Holman RR, on behalf of the UK Prospective Diabetes Study Group (2000). Association of glycaemia with macrovascular and microvascular complications of type 2 diabetes (UKPDS 35): prospective observational study. BMJ.

[119] Gomes MB, de Mattos Matheus AS, Calliari LE, Luescher JL, Manna TD, Savoldelli RD, Cobas RA, Coelho WS, Tschiedel B, Ramos AJ, Fonseca RM, Araujo NB, Almeida HG, Melo NH, Jezini DL, Negrato CA (2013). Economic status and clinical care in young type 1 diabetes patients: a nationwide multicenter study in Brazil. Acta Diabetol.

[120] Gomes MB, Giannella-Neto D, Faria M, Tambascia MA, Fonseca RM, Rea RR, Macedo G, Modesto Filho J, Schmid H, Bittencourt AV, Cavalcanti S, Rassi N, Pedrosa H, Atala Dib S (2006). Prevalence of type 2 diabetic patients within the targets of care guidelines in daily clinical practice: a multicenter study in Brazil. Rev Diabet Stud.

[121] American Diabetes Association (2008). Economic costs of diabetes in the US in 2007. Diabetes Care.

[122] Cobas RA, Ferraz MB, Matheus ASM, Tannus LRM, Negrato CA, Araújo LA, Dib SA, Gomes MB, Brazilian Type 1 Diabetes Study Group (2013). The cost of type 1 diabetes: a nationwide multicentre study in Brazil. Bull World Health Organ.

[123] Ceriello A, Sudhesh K, Piconi L, Esposito K, Giugliano D (2007). Simultaneous control of hyperglycemia and oxidative stress normalizes endothelial function in type 1 diabetes. Diabetes Care.

[124] Orchard TJ, Olson JC, Erbey JR, Williams K, Forrest KY, Smithline Kinder L, Ellis D, Becker DJ (2003). Insulin resistance-related factors, but not glycemia, predict coronary artery disease in type 1 diabetes. Diabetes Care.

[125] Gomes MB, Cobas RA, Nunes E, Castro-Faria-Neto HC, da Matta MF, Neves R (2009). Plasma PAF–acetylhydrolase activity, inflammatory markers and susceptibility of LDL to in vitro oxidation in patients with type 1 diabetes mellitus. Diabetes Res Clin Pract.

[126] Grundy SM, Benjamin IJ, Burke GL, Chait A, Eckel RH, Howard BV, Mitch W, Smith SC, Sowers JR (1999). Diabetes and cardiovascular disease: a statement for healthcare professionals from the American Heart Association. Circulation.

[127] van Vliet M, Van der Heyden JC, Diamant M, Von Rosenstiel IA, Schindhelm RK, Aanstoot HJ, Veeze HJ (2010). Overweight is highly prevalent in children with type 1 diabetes and associates with cardiometabolic Risk. J Pediatr.

[128] Evans JL, Goldfine ID, Maddux BA, Grodsky GM (2003). Are oxidative stress-activated signaling pathways mediators of insulin resistance and beta-cell dysfunction?. Diabetes.

[129] Chang YC, Chuang LM (2010). The role of oxidative stress in the pathogenesis of type 2 diabetes: from molecular mechanism to clinical implication. Am J Transl Res.

[130] Singh R, Barden A, Mori T, Beilin L (2001). Advanced glycation end- products: a review. Diabetologia.

[131] Giugliano D, Ceriello A, Paolisso G (1996). Oxidative stress and diabetic vascular complications. Diabetes Care.

[132] Ruderman NB, Williamson JR, Brownlee M (1992). Glucose and diabetic vascular disease. FASEB J.

[133] Di Mario U, Pugliese G (2001). 15th Golgi lecture: from hyperglycaemia to the dysregulation of vascular remodelling in diabetes. Diabetologia.

[134] Udupa AS, Nahar PS, Shah SH, Kshirsagar MJ, Ghongane BB (2012). Study of comparative effects of antioxidants on insulin sensitivity in type 2 diabetes mellitus. J Clin Diagn Res.

[135] Porasuphatana S, Suddee S, Nartnampong A, Konsil J, Harnwong B, Santaweesuk A (2012). Glycemic and oxidative status of patients with type 2 diabetes mellitus following oral administration of alpha- lipoic acid: a randomized double-blinded placebo-controlled study. Asia Pac J Clin Nutr.

[136] de Oliveira AM, Rondó PH, Luzia LA, D’Abronzo FH, Illison VK (2011). The effects of lipoic acid and α-tocopherol supplementation on the lipid profile and insulin sensitivity of patients with type 2 diabetes mellitus: a randomized, double-blind, placebo-controlled trial. Diabetes Res Clin Pract.

[137] Santos JM, Kowluru RA (2011). Role of mitochondria biogenesis in the metabolic memory associated with the continued progression of diabetic retinopathy and its regulation by lipoic acid. Invest Ophthalmol Vis Sci.

[138] Lin J, Bierhaus A, Bugert P, Dietrich N, Feng Y, Vom Hagen F, Nawroth P, Brownlee M, Hammes HP (2006). Effect of R-(+)-alpha-lipoic acid on experimental diabetic retinopathy. Diabetologia.

[139] Haritoglou C, Gerss J, Hammes HP, Kampik A, Ulbig MW, RETIPON Study Group (2011). Alpha-lipoic acid for the prevention of diabetic macular edema. Ophthalmologica.

[140] Nebbioso M, Federici M, Rusciano D, Evangelista M, Pescosolido N (2012). Oxidative stress in preretinopathic diabetes subjects and antioxidants. Diabetes Technol Ther.

[141] Yi X, Xu L, Hiller S, Kim HS, Nickeleit V, James LR, Maeda N (2012). Reduced expression of lipoic acid synthase accelerates diabetic nephropathy. J Am Soc Nephrol.

[142] Bhatti F, Mankhey RW, Asico L, Quinn MT, Welch WJ, Maric C (2005). Mechanisms of antioxidant and pro-oxidant effects of alpha-lipoic acid in the diabetic and nondiabetic kidney. Kidney Int.

[143] Borcea V, Nourooz-Zadeh J, Wolff SP, Klevesath M, Hofmann M, Urich H, Wahl P, Ziegler R, Tritschler H, Halliwell B, Nawroth PP (1999). alpha- Lipoic acid decreases oxidative stress even in diabetic patients with poor glycemic control and albuminuria. Free Radic Biol Med.

[144] Cicek M, Yıldırır A, Okyay K, Yazici AC, Aydinalp A, Kanyilmaz S, Muderrisoglu H (2013). Use of alpha-lipoic acid in prevention of contrast- induced nephropathy in diabetic patients. Ren Fail.

[145] Chang JW, Lee EK, Kim TH, Min WK, Chun S, Lee KU, Kim SB, Park JS (2007). Effects of alpha-lipoic acid on the plasma levels of asymmetric dimethylarginine in diabetic end-stage renal disease patients on hemodialysis: a pilot study. Am J Nephrol.

[146] Morcos M, Borcea V, Isermann B, Gehrke S, Ehret T, Henkels M, Schiekofer S, Hofmann M, Amiral J, Tritschler H, Ziegler R, Wahl P, Nawroth PP (2001). Effect of alpha-lipoic acid on the progression of endothelial cell damage and albuminuria in patients with diabetes mellitus: an exploratory study. Diabetes Res Clin Pract.

[147] Heinisch BB, Francesconi M, Mittermayer F, Schaller G, Gouya G, Wolzt M, Pleiner J (2010). Alpha-lipoic acid improves vascular endothelial function in patients with type 2 diabetes: a placebo-controlled randomized trial. Eur J Clin Invest.

[148] Chen SA, Chen HM, Yao YD, Hung CF, Tu CS, Liang YJ (2012). Topical treatment with anti-oxidants and Au nanoparticles promote healing of diabetic wound through receptor for advance glycation end-products. Eur J Pharm Sci.

[149] Ziegler D, Schatz H, Conrad F, Gries FA, Ulrich H, Reichel G (1997). Effects of treatment with the antioxidant alpha-lipoic acid on cardiac autonomic neuropathy in NIDDM patients. A 4-month randomized controlled multicenter trial (DEKAN Study). Deutsche Kardiale Autonome Neuropathie. Diabetes Care.

[150] Pop-Busui R, Stevens MJ, Raffel DM, White EA, Mehta M, Plunkett CD, Brown MB, Feldman EL (2013). Effects of triple antioxidant therapy on measures of cardiovascular autonomic neuropathy and on myocardial blood flow in type 1 diabetes: a randomised controlled trial. Diabetologia.

[151] Ziegler D (1996). Diagnosis and mangement of diabetic peripheral neuropathy. Diabet Med.

[152] Dyck PJ, Davies JL, Litchy WJ, O’Brien PC (1997). Longitudinal assessment of diabetic polyneuropathy using a composite score in the Rochester Diabetic Neuropathy Study cohort. Neurology.

[153] Ziegler D, Hanefeld M, Ruhnau KJ, Meissner HP, Lobisch M, Schütte K, Gries FA (1995). Treatment of symptomatic diabetic peripheral neuropathy with the anti-oxidant alpha-lipoic acid. A 3-week multicentre randomized controlled trial (ALADIN Study). Diabetologia.

[154] Reljanovic M, Reichel G, Rett K, Lobisch M, Schuette K, Möller W, Tritschler HJ, Mehnert H (1999). Treatment of diabetic polyneuropathy with the antioxidant thioctic acid (alpha-lipoic acid): a two year multicenter randomized double-blind placebo-controlled trial (ALADIN II). Alpha Lipoic Acid in Diabetic Neuropathy. Free Radic Res.

[155] Ziegler D, Hanefeld M, Ruhnau KJ, Hasche H, Lobisch M, Schütte K, Kerum G, Malessa R (1999). Treatment of symptomatic diabetic polyneuropathy with the antioxidant alpha-lipoic acid: a 7-month multicenter randomized controlled trial (ALADIN III Study). ALADIN III Study Group. Alpha-Lipoic Acid in Diabetic Neuropathy. Diabetes Care.

[156] Ruhnau KJ, Meissner HP, Finn JR, Reljanovic M, Lobisch M, Schütte K, Nehrdich D, Tritschler HJ, Mehnert H, Ziegler D (1999). Effects of 3-week oral treatment with the antioxidant thioctic acid (alpha-lipoic acid) in symptomatic diabetic polyneuropathy. Diabet Med.

[157] Ametov AS, Barinov A, Dyck PJ, Hermann R, Kozlova N, Litchy WJ, Low PA, Nehrdich D, Novosadova M, O’Brien PC, Reljanovic M, Samigullin R, Schuette K, Strokov I, Tritschler HJ, Wessel K, Yakhno N, Ziegler D, SYDNEY Trial Study Group (2003). The sensory symptoms of diabetic polyneuropathy are improved with alpha-lipoic acid: the SYDNEY trial. Diabetes Care.

[158] Ziegler D, Ametov A, Barinov A, Dyck PJ, Gurieva I, Low PA, Munzel U, Yakhno N, Raz I, Novosadova M, Maus J, Samigullin R (2006). Oral treatment with alpha-lipoic acid improves symptomatic diabetic polyneuropathy: the SYDNEY 2 trial. Diabetes Care.

[159] Ziegler D, Low PA, Litchy WJ, Boulton AJ, Vinik AI, Freeman R, Samigullin R, Tritschler H, Munzel U, Maus J, Schütte K, Dyck PJ (2011). Efficacy and safety of antioxidant treatment with α-lipoic acid over 4 years in diabetic polyneuropathy: the NATHAN 1 trial. Diabetes Care.

[160] Ziegler D, Nowak H, Kempler P, Vargha P, Low PA (2004). Treatment of symptomatic diabetic polyneuropathy with the antioxidant alpha-lipoic acid: a meta-analysis. Diabet Med.

[161] Mijnhout GS, Kollen BJ, Alkhalaf A, Kleefstra N, Bilo HJ (2012). Alpha lipoic acid for symptomatic neuropathy in patients with diabetes: A meta-analysis of randomized controlled trials. Int J Endocrinol.

[162] Bertolloto F, Massome A (2012). Combination of alpha lipoic acid and superoxide dismutase leads to physiological and symptomatic improvements in diabetic neuropathy. Drugs.

[163] Vasudevan D, Naik MM, Mukaddam QI (2014). Efficacy and safety of methylcobalamin, alpha lipoic acid and pregabalin combination versus pregabalin monotherapy in improving pain and nerve conduction velocity in type 2 diabetes associated impaired peripheral neuropathic condition. Results of a pilot study. Ann Indian Acad Neurol.

[164] Patel N, Mishra V, Patel P, Dikshot RK (2014). A study of the use of carbamazepine, pregabalin and alpha lipoic acid in patients of diabetic neuropathy. J Diabetes Metab Disord.

[165] Bresciani E, Busi A, Bazzigaluppi E, Balestere G (2011). Insulin autoimmune syndrome induced by α lipoic acid in a Caucasian woman: case report. Diabetes Care.

[166] Mooradian AD, Haas MJ (2011). Glucose-induced endoplasmic reticulum stress is independent of oxidative stress: a mechanistic explanation for the failure of antioxidant therapy in diabetes. Free Radic Biol Med.

