# Supplementary figures and images for: Alpha-lipoic acid as a pleiotropic compound with potential therapeutic use in diabetes and other chronic diseases
Source: Diabetol Metab Syndr. 2014 Jul 28;6:80. doi: 10.1186/1758-5996-6-80 (PMC4124142; doi:10.1186/1758-5996-6-80)

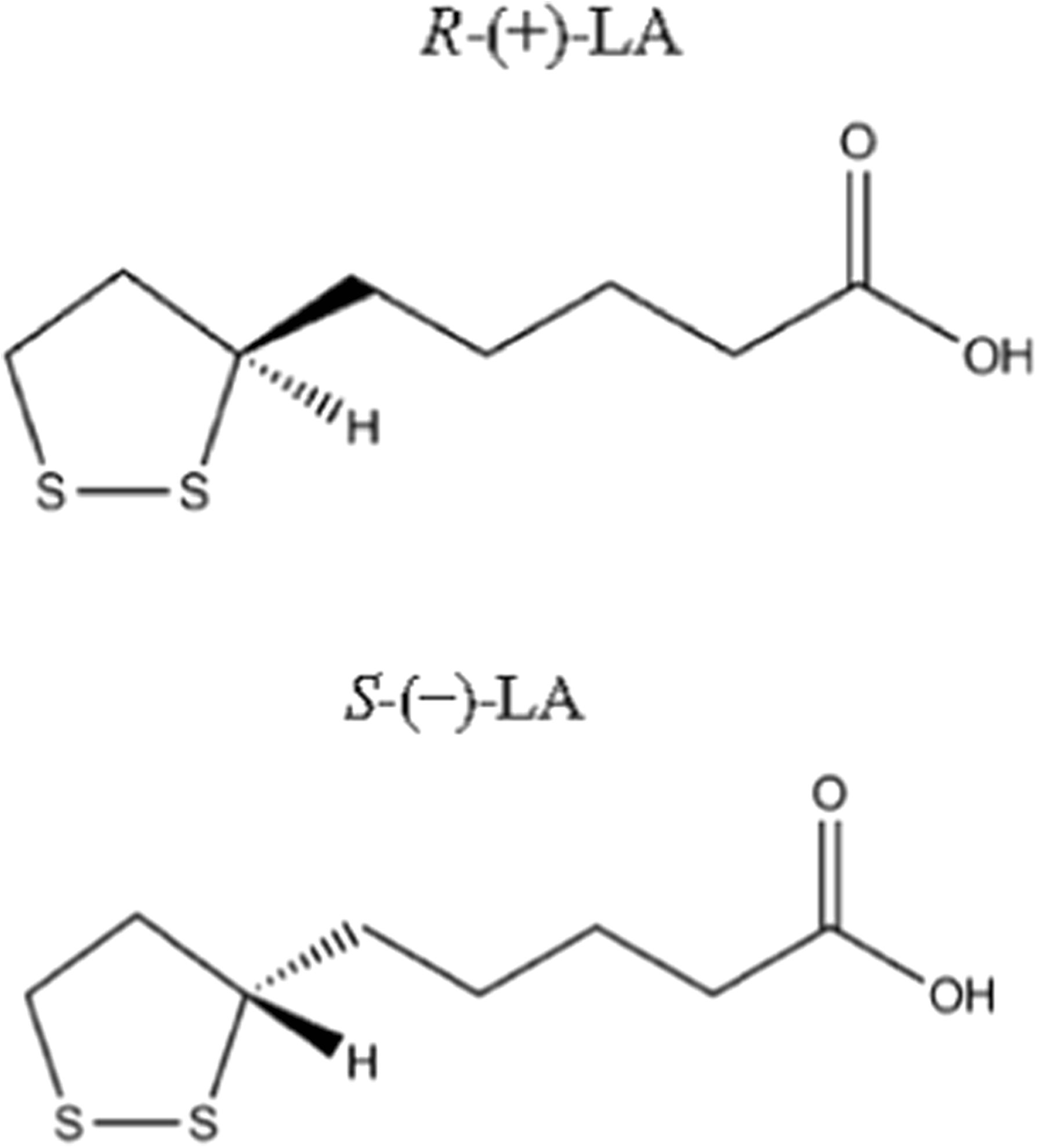

Supplement: Supplementary file 1 — Authors’ original file for figure 1 [file 13098_2014_345_MOESM1_ESM.tif]

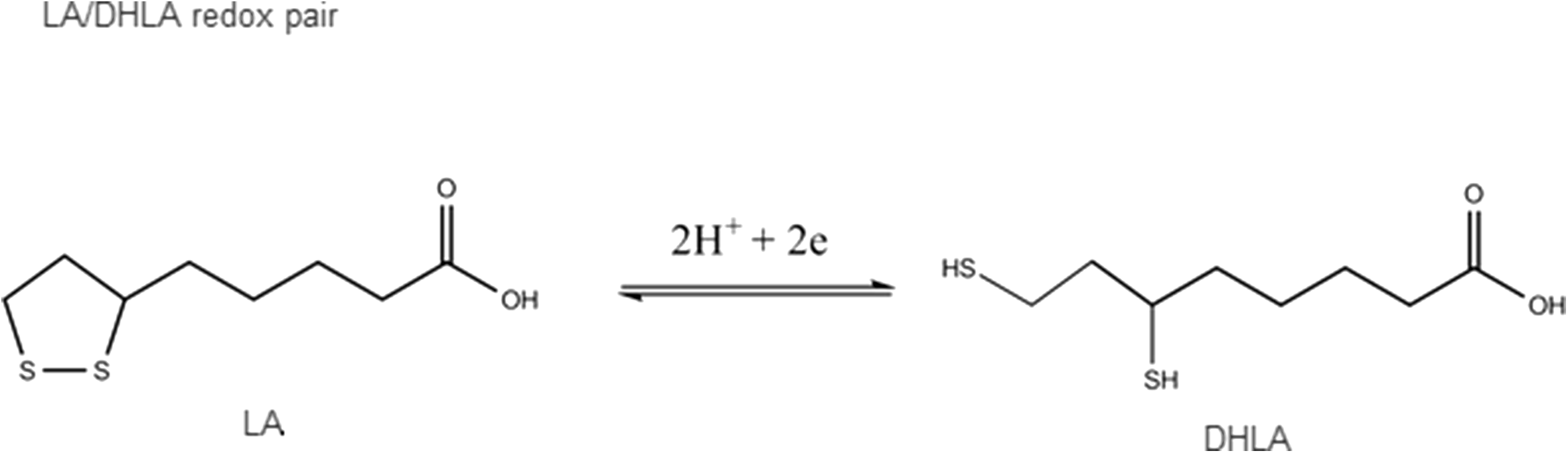

Supplement: Supplementary file 2 — Authors’ original file for figure 2 [file 13098_2014_345_MOESM2_ESM.tif]
